# Synthesis, and Structural and Spectroscopic Analysis of Trielyl‐Derived Complexes of Iron

**DOI:** 10.1002/chem.202404451

**Published:** 2025-03-05

**Authors:** Liam P. Griffin, Alexis K. Bauer, Agamemnon E. Crumpton, Mathias A. Ellwanger, Andreas Heilmann, Anja Wiesner, Michael L. Neidig, Simon Aldridge

**Affiliations:** ^1^ Inorganic Chemistry Laboratory Department of Chemistry University of Oxford South Parks Road Oxford OX1 3QR UK; ^2^ Institute of Inorganic Chemistry Freie Universität Berlin Fabeckstr. 34/36 14195 Berlin Germany

**Keywords:** aluminium, gallium, indium, iron, Mössbauer spectroscopy

## Abstract

The reactivity of group 13 anions of the form [(NON)E]^−^ (NON=4,5‐bis(2,6‐diisopropylanilido)‐2,7‐di‐*tert*‐butyl‐9,9‐dimethyl‐xanthene, E=Al, Ga, In) towards Fe(CO)_5_ has been investigated. In the case of the aluminyl system, both reaction outcome and product structure are highly sensitive to the availability of the potassium counterion; sequestration by 18‐crown‐6 is necessary to yield a species featuring a direct, unsupported Al−Fe bond. 2.2.2‐Cryptand, by contrast, yields a species featuring bridging carbonyl ligands, while the use of no sequestering agent at all leads to isocarbonyl bridging to aluminium. Owing to their lower oxophilicity, the heavier congeners gallium and indium more straightforwardly deliver Fe−E bonded adducts (E=Ga, In). The series of trielyl iron complexes has been interrogated by structural and computational analyses, as well as by IR and Mössbauer spectroscopies, revealing a consistent shift in bond polarity and electron richness at iron as group 13 is descended. This in turn is consistent with the diminishing donor strength of the trielyl ligand with increasing atomic number.

## Graphical abstract

Iron aluminyl‐, gallyl‐ and indyl‐derived complexes of the type [(NON)EFe(CO)_4_]^−^ (E=Al, Ga, In; NON=4,5‐bis(2,6‐diisopropylanilido)‐2,7‐di‐*tert*‐butyl‐9,9‐dimethyl‐xanthene) have been synthesized, and the properties of the group 13 metallo‐ligands compared by a combination of crystallographic, computational and IR/Mössbauer spectroscopic techniques.

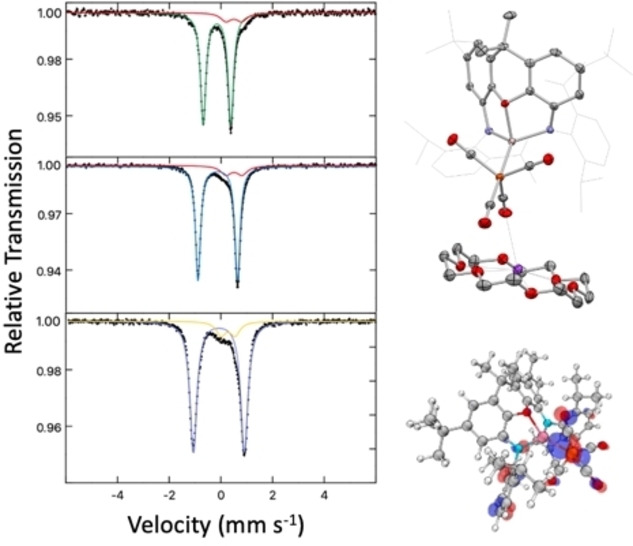

## Introduction

The synthesis of metal complexes bearing the heavier group 13 analogues of boryl ligands (i. e. EX_2_^−^, where E=Al−Tl) has been an significant target for synthetic chemists in recent years.[^1^, ^2^, ^3^, ^4^] Metal boryl complexes themselves (L_*n*_MBX_2_) have shown great utility in the C−H functionalization/borylation of unactivated alkane and arene substrates, in part due to the combination of strong σ‐donor capabilities and (albeit modest) Lewis acidic properties of the boryl moiety.^[5]^ The synthesis of heavier trielyl complexes not only offers fundamental insight into the geometric and electronic properties of these metallo‐ligands in comparison with their boron analogues, but also promises new small molecule transformations enabled across the metal‐metal bonds of transition metal‐triel bimetallics.

In the case of aluminium, its low electronegativity and high oxophilicity, partnered with the strongly reducing nature of Al(I) suggest that metal complexes featuring the aluminyl ligand might possess unusual electronic properties (and reactivity profiles) as a result of the close proximity of highly electron rich and electron deficient metal centres.^[6]^ This scenario is amply demonstrated by the gold aluminyl complex ^t^Bu_3_PAuAl(NON) which reacts with CO_2_ through the formation of Au−C and Al−O bonds, in a process involving the gold centre formally acting as a nucleophile.^[7]^ Given that trielyl ligands are now available for all of the group 13 metals (E=Al−Tl), a fundamental understanding of electronic structure as a function of E offers a mechanism through which to tune such reactivity.[^8^, ^9^, ^10^, ^11^]

**Figure 1:**
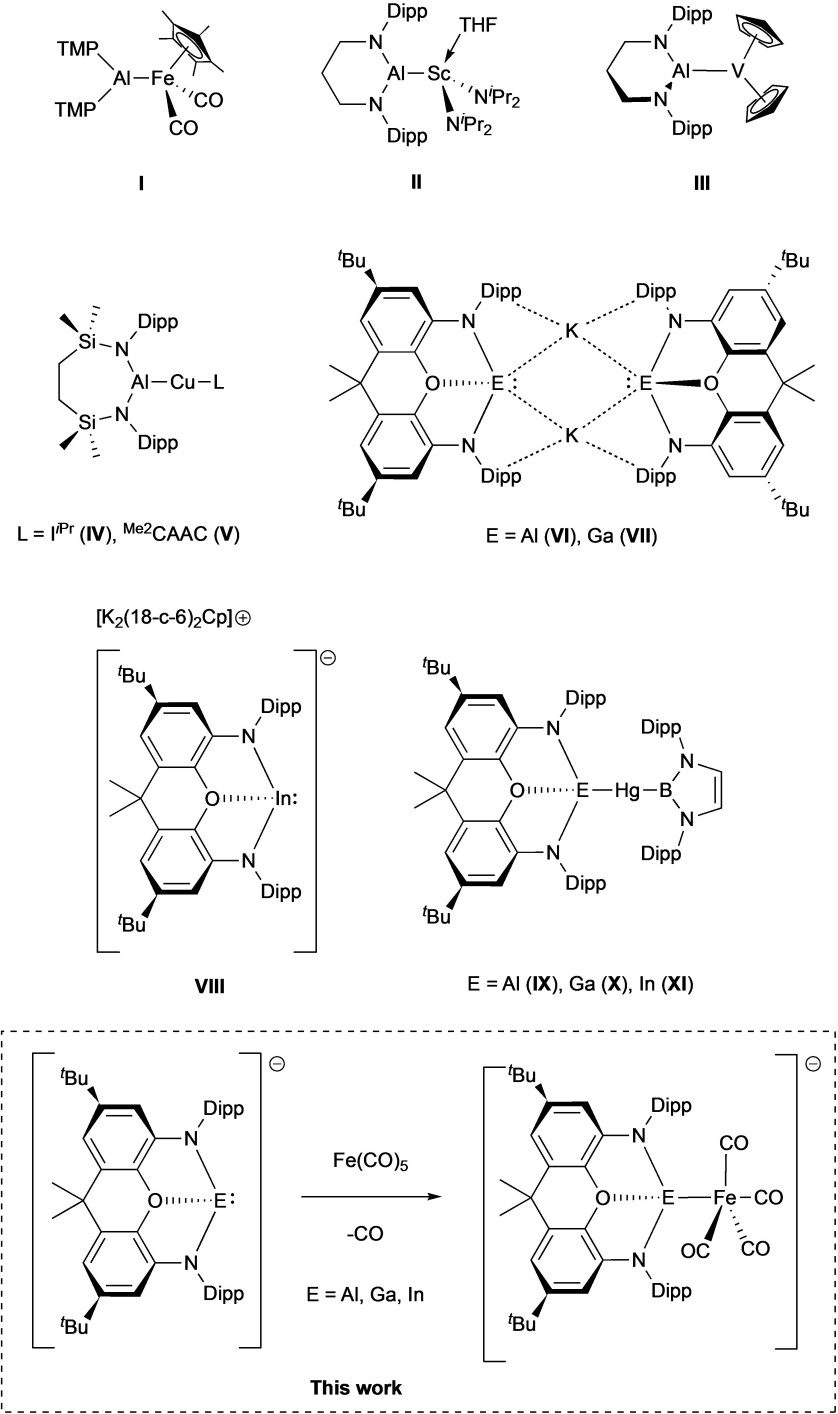
Key compounds of relevance to this study. Representative iron aluminyl complexes of 3d metals (**I**–**V**); aluminium, gallium and indium (I) anions stabilized by a common xanthene ligand framework (**VI**–**VIII**); an isostructural series of (boryl)mercury trielyl complexes generated from nucleophilic trielyl reagents (**IX**–**XI**). This work: accessing an essentially isostructural series of iron carbonyl complexes of trielyl ligands (Dipp=2,6‐^*i*^Pr_2_C_6_H_3_).

In terms of synthesis, early examples of complexes bearing aluminyl ligands were accessed either by reduction or Al−X oxidative addition at heterobimetallic systems bearing bridging ligands (to generate supported M−Al bonds) or, for transition metals for which suitably nucleophilic precursors exist (typically containing π‐acceptor ligands, such as carbonyls), via salt metathesis at an electrophilic Al(III) halide. By far the most common example of this approach involves the use of the [CpFe(CO)_2_]^−^ anion, due to its relative ease of synthesis and handling (e. g. **I**, Figure 1).[^12^, ^13^, ^14^] Approaches that make use of low valent aluminium precursors have also been known for several years, with the oxidative addition of M−X bonds at a charge neutral Al(I) centre such as that in (Nacnac^Dipp^)Al (Nacnac^Dipp^=HC(MeCDippN)_2_; Dipp=2,6‐C_6_H_3_^*i*^Pr_2_) yielding metal aluminyl species for metals such as beryllium and copper.[^15^, ^16^]

Since the first report of a potassium aluminyl species in 2018, the range of accessible M−Al bonds has increased greatly; isolable nucleophilic precursors of the type [AlX_2_]^−^ can (in theory) be combined with a wide range of metal electrophiles, and this approach has been used to synthesise complexes featuring metals from groups 1, 2, 3, 5, 10, 11 and 12 (as well as the rare earth metal samarium) (e. g. **II**–**V**).[^8^, ^17^, ^18^, ^19^, ^20^, ^21^, ^22^, ^23^, ^24^, ^25^, ^26^, ^27^, ^28^] Unsurprisingly, aluminyl species were the last of the group 13 element trieyl derivatives for which a simple alkali metal salt could be accessed, owing to the same elemental properties which make their application as ligands so interesting. In our laboratory, having access to a common series of otherwise equivalent aluminyl, gallyl and indyl metallo‐ligands (**VI**–**VIII**), we have been interested in probing trends in the properties of these systems for a common supporting scaffold (e. g. **IX**–**XI**). As part of these studies, we turned to the iron carbonyl fragment [Fe(CO)_4_], as a classic organometallic probe‐group, to deliver data on the properties of these metallo‐ligands at a *d*‐block metal centre through IR and Mossbauer spectroscopies, X‐ray crystallography and quantum chemical calculations. These studies are reported here.

## Results and Discussion

### Syntheses and Structures of Aluminyl‐Derived Complexes of Iron

**Scheme 1:**
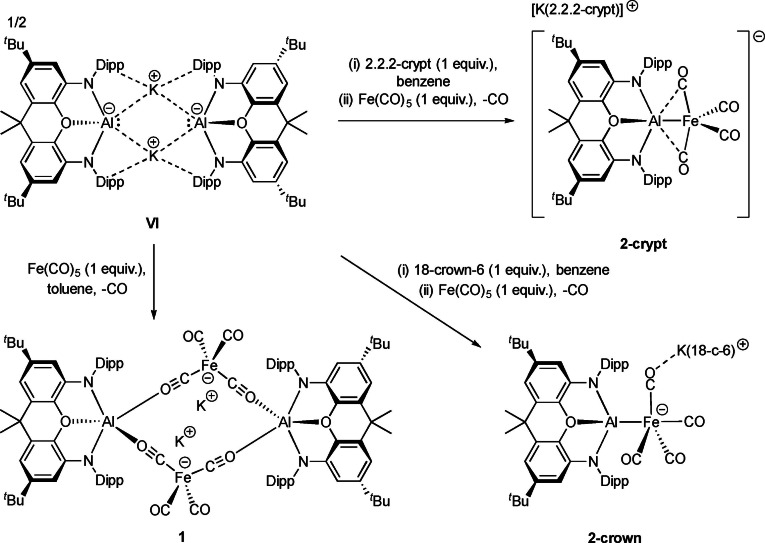
Reactivity of **VI** towards Fe(CO)_5_ in the presence or absence of potassium cation sequestering agents.

**Figure 2:**
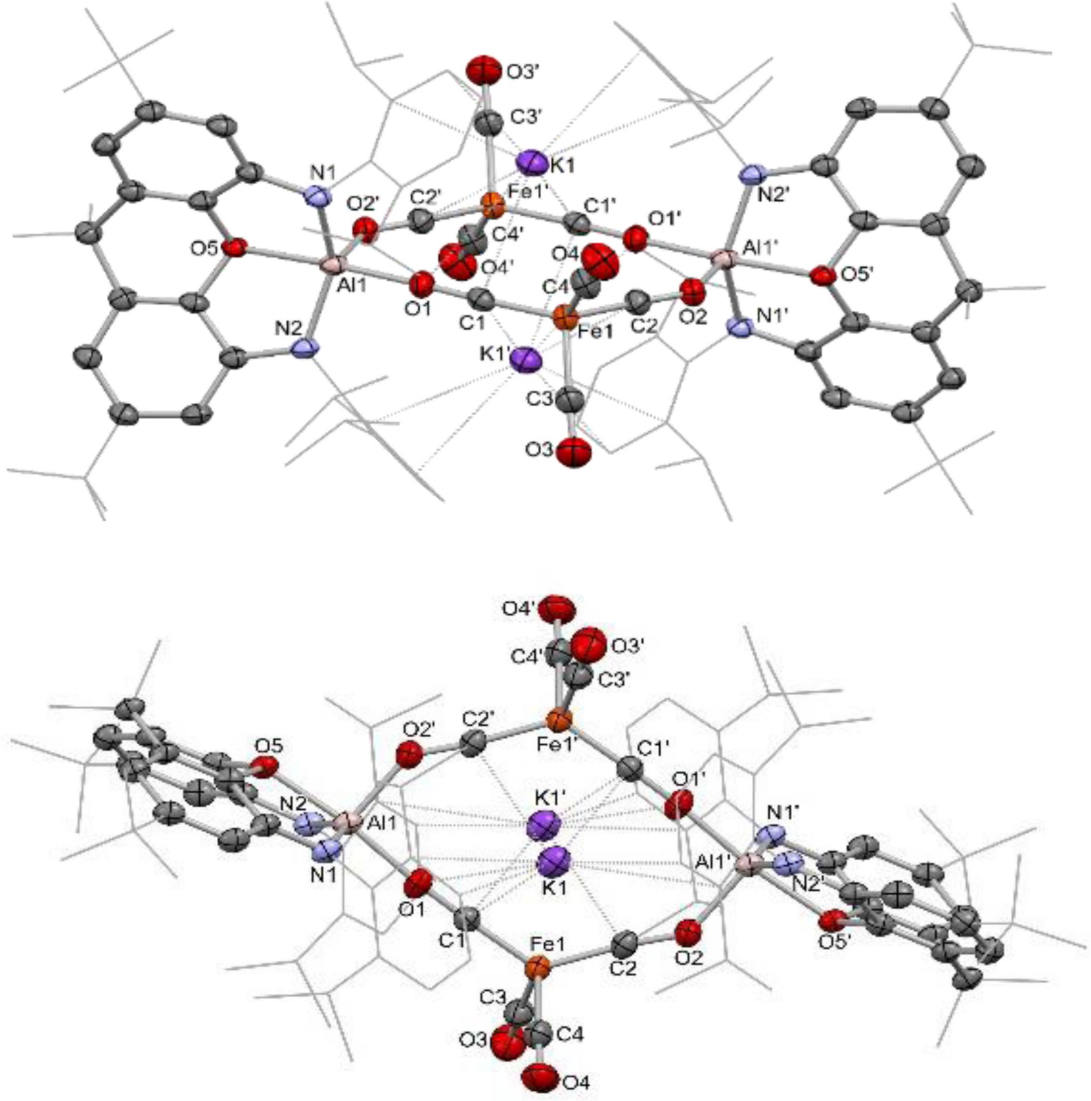
Molecular structure of **1**, as determined by X‐ray crystallography. H atoms and solvate molecule omitted, and some residues displayed in wireframe format for clarity. Key bond lengths (Å) and angles (°): Al1−O1 1.855(2), Al1−O5 1.984(2), Al1−N1 1.878(2), Al1−N2 1.872(2), Al1−O2’ 1.830(2), Fe1−C1 1.711(3), Fe1−C2 1.705(3), Fe1−C3 1.799(3), Fe1−C4 1.784(3), C1−O1 1.211(3), C2−O2 1.227(3), C3−O3 1.149(4), C4−O4 1.157(4), K1−O1 3.350(2), K1−C1 3.140(3), K1−C1’ 3.215(3), K1−C2’ 3.357(3), O1−Al1−O5 173.39(9), O1−Al1−O2’ 94.12(9), O5−Al1−O2’ 92.49(9), N1−Al1−N2 140.2(1), N1−Al1−O2’ 107.6(1), N2−Al1−O2’ 109.0(1), C1−Fe1−C2 126.0(1), C3−Fe1−C4 98.3(1).

Seeking to access iron trielyl complexes directly, the reactions between sources of [(NON)E]^−^ (E=Al, Ga, In) and Fe(CO)_5_ were targeted, via substitution of a single CO ligand (Scheme 1). Reaction between Fe(CO)_5_ and K_2_[(NON)Al]_2_ in toluene occurs immediately, yielding a dark solution, from which colourless crystals suitable for X‐ray crystallography could be grown by slow evaporation. However, crystallographic studies reveal that, rather than proceeding via Fe−Al bond formation, a dimeric species is generated in which two [Fe(CO)_4_] units are linked by a pair of [(NON)Al] fragments via isocarbonyl‐type ligation at the aluminium centres (Figure 2). The resulting structure features a 12‐membered metallo‐macrocyclic structure. This dianionic ring is capped above each face by a potassium cation, which is coordinated by the π‐systems of both the NON arene groups and the bridging isocarbonyl ligands. This species can also be viewed as a dimeric analogue of Collman's reagent, K_2_Fe(CO)_4_, in which one of the potassium cations per iron has been exchanged for a [(NON)Al^III^]^+^ cation.^[29]^ Related heterobimetallic isocarbonyl species are known for aluminium, as well as gallium and the oxophilic lanthanide elements.^[30]^

The crystallographically equivalent aluminium centres adopt a significantly distorted trigonal bipyramidal (TBP) geometry (τ_5_=0.55), in which one isocarbonyl O atom and the xanthene O atom are aligned approximately linearly (173.39(9)°), but feature disparate Al−O distances (1.855(2) and 1.984(2) Å, respectively). Distortion from an idealised TBP geometry reflects the wider angle defined by the N‐donors (N−Al−N 140.2(1)°) which also lie marginally out of the equatorial plane as a result of the constraints of the chelating xanthene framework. The iron centres each adopt a distorted tetrahedral geometry (τ_4_=0.89), with a wider angle between the two isocarbonyl ligands (126.0(1)°), and a narrower angle between the two conventional carbonyl ligands (98.3(1)°). A significant extension of the C−O bond is seen for the bridging isocarbonyl ligands as compared to the'conventional’ terminal CO ligands (isocarbonyls: 1.211(3) and 1.227(3) Å cf. 1.149(4) and 1.157(4) Å).

**Figure 3:**
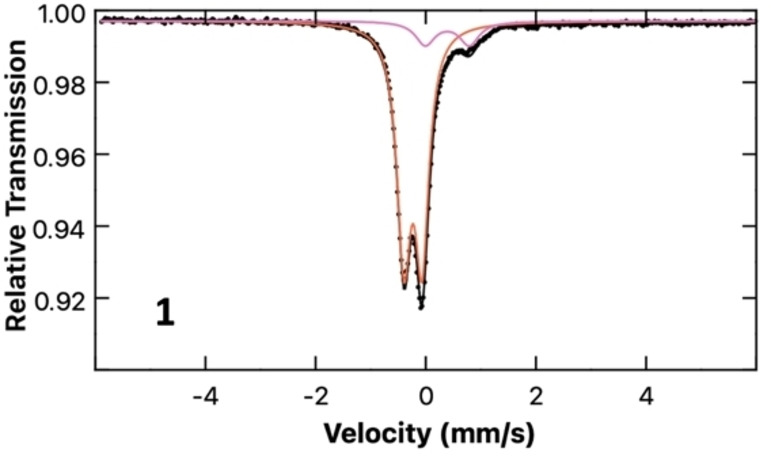
Zero field 80 K ^57^Fe Mössbauer spectrum of 1 (orange, 87 %): δ=−0.23 mm/s, |ΔE_Q_|=0.33 mm/s; (pink, 13 %): δ=0.39 mm/s, |ΔE_Q_|=0.80 mm/s.

The description of this species as a Collman's reagent analogue is supported by computational analysis. Bader's Quantum Theory of Atoms In Molecules (QT‐AIM) charge analysis assigns a charge of −1.78 e to the [Fe(CO)_4_] fragment, +0.88 e to the K atoms, and +0.90 e to the (NON)Al unit. The aluminium atom itself is assigned a charge of +2.55 e, and this highly polarising cation is, in turn, presumably responsible from the non‐uniform distribution of negative charge in the [Fe(CO)_4_]^2−^ unit. This polarisation can be seen in the individual CO charge contributions, which become more negative on moving from the terminal carbonyls (−0.30 and −0.28 e) to the bridging isocarbonyls (−0.89 and −0.89 e). A Mössbauer spectrum measured for a solid‐state sample of **1** (Figure 3) supports the DFT analysis, based on the negative shift δ=−0.23 mm/s and quadrupole splitting |ΔE_Q_|=0.33 mm/s, which are consistent with a highly reduced iron species.^[31]^ The minor impurity identified in the spectrum, characterized by δ=0.39 mm/s and |ΔE_Q_|=0.80 mm/s, resembles the reported parameters for Fe_3_(CO)_12_ (δ=0.30 mm/s; |ΔE_Q_|=0.98 mm/s).^[32]^ Infrared measurements yield CO stretching frequencies of 1906 and 1970 cm^−1^, which align closely with the *T*_2_ stretching band of Collman's reagent (1972 cm^−1^).

**Figure 4:**
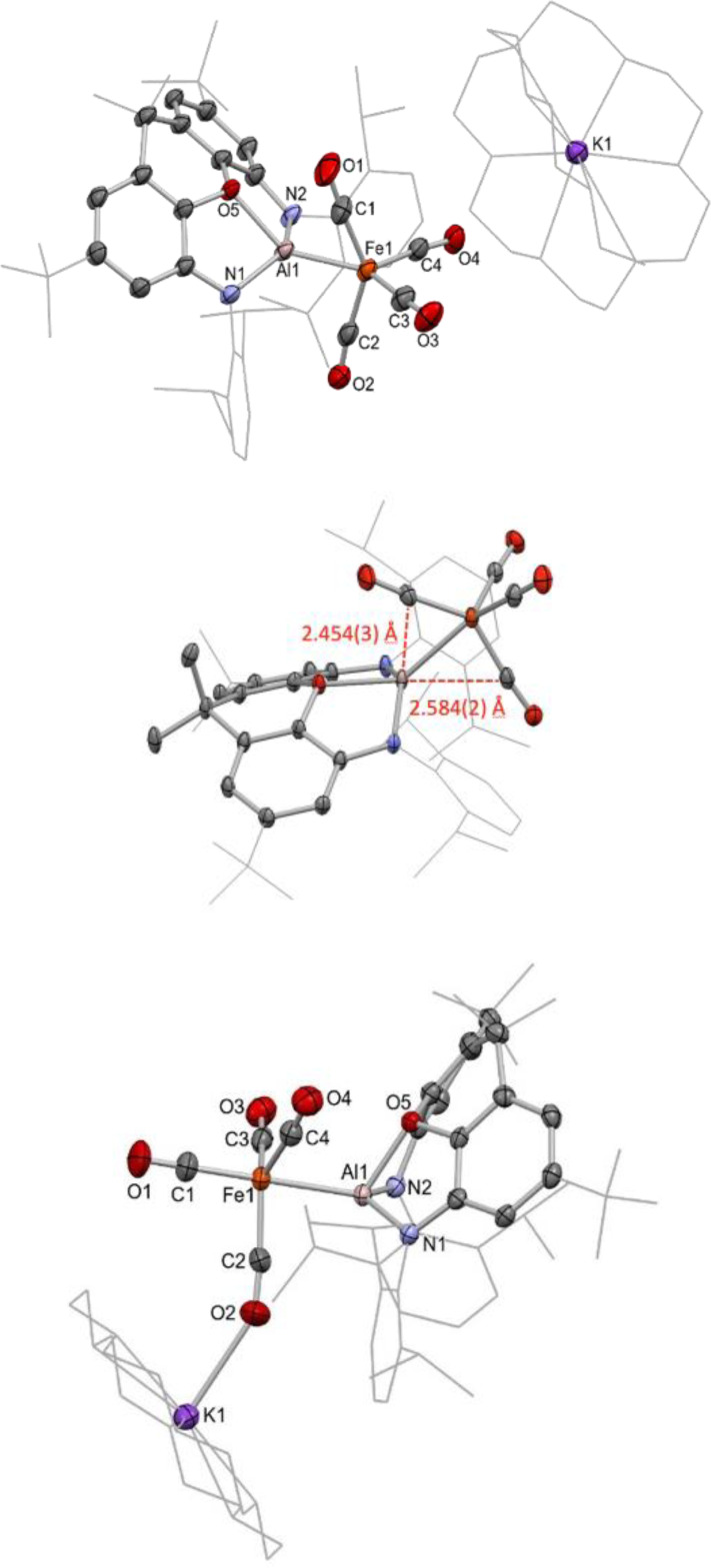
Molecular structures of **2**‐crypt (upper and centre) and **2**‐crown (lower), as determined by X‐ray crystallography. H atoms and solvate molecule omitted, and some residues displayed in wireframe format for clarity. Key bond lengths (Å) and angles (°): (for **2‐crypt**) Al1−Fe1 2.3938(7), Al1−O5 2.029(1), Al1−N1 1.920(2), Al1−N2 1.901(2), Al1−C1 2.454(3), Al1−C2 2.584(2), Fe1−C1 1.754(2), Fe1−C2 1.764(3), Fe1−C3 1.769(2), Fe1−C4 1.777(2), C1−O1 1.174(3), C2−O2 1.168(3), C3−O3 1.159(5), C4−O4 1.154(3), Al1−Fe1−C1 70.63(9), Al1−Fe1−C2 75.15(8), Al1−Fe1−C3 147.30(8), Al1−Fe1−C4 113.69(7), C1−Fe1−C2 141.2(1), C3−Fe1−C4 99.0(1). (for **2‐crown**) Al1−Fe1 2.374(1), Al1−O5 1.973(2), Al1−N1 1.888(3), Al1−N2 1.904(3), Fe1−C1 1.778(4), Fe1−C2 1.759(4), Fe1−C3 1.781(4), Fe1−C4 1.767(4), C1−O1 1.146(6), C2−O2 1.160(5), C3−O3 1.152(5), C4−O4 1.151(5), O2−K1 2.731(4), Al1−Fe1−C1 174.2(1), Al1−Fe1−C2 82.8(2), C1−Fe1−C2 96.8(2), C2−Fe1−C3 121.3(2), C2−Fe1−C4 121.5(2), C3−Fe1−C4 112.2(2).

At a broader level, the solid‐state structure of **1** raises the question of whether the capping potassium ions are significant in stabilizing this geometric motif, and if K^+^ sequestration prior to addition of Fe(CO)_5_ might influence the outcome of the reaction. Literature precedent for reactivity tuning based on the identity/availability of the alkali‐metal counterion is well established, but has yet to be investigated in the context of bimetallic compounds.[^17^, ^33^, ^34^] With this in mind, the reaction between [K(2.2.2‐crypt][(NON)Al] and Fe(CO)_5_ was investigated. Here too, reaction occurs instantaneously and yields a dark solution, from which a small quantity of single crystals of [K(2.2.2‐crypt)][(NON)AlFe(CO)_4_] (**2‐crypt**) suitable for X‐ray diffraction experiments could be obtained (Scheme 1 and Figure 4).

Crystallographic analysis reveals that sequestration of the potassium cation by the cryptand does indeed alter the course of the reaction, in this case yielding a metal‐metal bonded species (albeit in low yield), partnered by a well‐separated [K(2.2.2‐crypt)]^+^ counterion. The monoanionic [(NON)AlFe(CO)_4_]^−^ fragment (Figure 4) features an Fe*−*Al bond length (2.3938(7) Å) that is well within the sum of the respective covalent radii of (2.53 Å).[^35^, ^36^] The geometry at the iron centre, however, is significantly distorted from the TBP expected for an [Fe(CO)_4_X]^−^ system, most obviously in the position of two CO ligands (C1O1 and C2O2). These are aligned such that the carbon atoms sit close to the axial and equatorial sites within the TBP coordination sphere of Al(1) commonly seen for [(NON)AlX_2_]^−^ systems (*d*(Al1−C1)=2.454(3) Å; *d*(Al1−C2) 2.584(2) Å). This distortion presumably results from the highly polarizing nature of the aluminium centre, which has offloaded a significant portion of its electron density to the iron carbonyl fragment (see below), as well as from the lack of an available potassium counterion to provide partial charge neutralization through isocarbonyl coordination. Returning to the coordination environment at iron, the geometry defined by the carbonyl ligands alone is reasonably close to tetrahedral (τ_4_=0.78), with one expanded angle (C1−Fe1−C2 141.2(1)°) and one contracted angle (C3−Fe1−C4 99.0(1)°), indicating that, here too, an overall reduction to a species akin to Collman's reagent has taken place, at least insofar as formal oxidation states are concerned. In this formalism, the [(NON)Al]^+^ moiety acts as a Z‐type ligand at a dianionic iron fragment, being bound through Fe→Al and C→Al donor/acceptor interactions. A Bader charge analysis supports this description, returning charges of −1.62 e for the Fe(CO)_4_ unit, and +0.62 e for the (NON)Al fragment. The Al centre is again highly polarizing, with the proximal CO ligands bearing significantly more negative charge (−0.62 and −0.51 e) compared to the remote carbonyls (−0.32 and −0.31 e).

**Figure 5:**
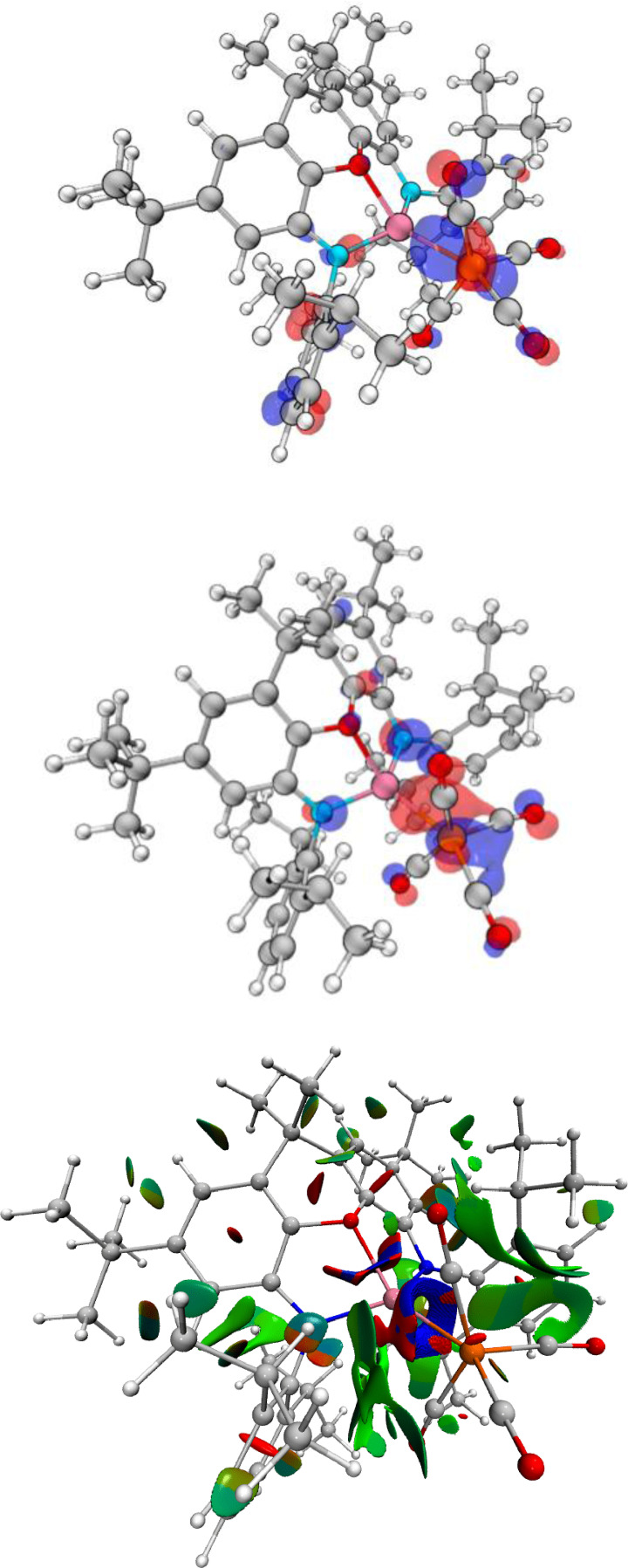
HOMO‐1 (upper) and HOMO‐6 (centre) of **2‐crypt**, showing the Al−Fe bonding interaction as well as involvement of CO π* density. (lower) NCI plot of **2‐crypt**, showing strong attractive regions (blue) between the aluminium centre and the proximal carbonyl carbon atoms C1 and C2.

DFT calculations for the free [(NON)AlFe(CO)_4_]^−^ anion return an optimised geometry which matches the crystallographic data well (see ESI). The HOMO of this species is an iron centred lone pair, which is significantly delocalised into the (π‐acceptor) carbonyl manifold; the LUMO is xanthene ligand based. A significant Al−Fe σ‐bonding contribution is seen in the HOMO‐1, with clear involvement of the CO π* manifold of a proximal CO ligand. Further interactions between the aluminium centre and the occupied π* orbitals of the other carbonyl ligands (including one of the distal carbonyls) are seen in the HOMO‐5 and HOMO‐6 (Figure 5). A QT‐AIM topological analysis of [(NON)AlFe(CO)_4_]^−^ returns a Bond Critical Point (BCP) between iron and aluminium, with associated parameters that indicate metallic‐type bonding. No bond paths are detected between aluminium and the two nearest carbonyl ligands, despite Wiberg Bond Indices (WBIs) for these atom pairs being of similar magnitude to that of the Fe−Al bond (Fe−Al 0.34; Al−C1 0.37; Al−C2 0.33). However, inspection of the Non‐Covalent Interactions (NCI) plot for this species locates these interactions, with significant attractive density between these carbon atoms and the aluminium centre (Figure 5).

With a view to accessing an Fe−Al bonded species in higher yield, and to further explore the effects of counterion availability, the corresponding reaction utilising 18‐crown‐6 was attempted (Scheme 1). The reaction is again very rapid and yields a substantial crop of colourless rod‐shaped crystals suitable for X‐ray diffraction. The crystal structure of this species, **2‐crown**, also features a direct Fe−Al bond, but this time within a more conventional TBP iron coordination sphere (τ_5_=0.88), with the strongly σ‐donating aluminyl ligand occupying the expected axial position (Figure 4). The angle between the two axial ligands at iron (∠Al1−Fe1−C1 174.2(1)°) is approximately linear, and the equatorial ligands sit relatively close to the ideal plane (∠Al1−Fe1−C2 82.8(2)°; ∠C1−Fe1−C2=96.8(2)°; sum of angles in the equatorial plane 355.0°). The more open nature of the crown ether ligand (cf. 2.2.2‐cryptand) allows access to the potassium cation by the oxygen atom of an equatorial CO ligand (in the solid state at least). This cation effect is structurally significant, insofar as it appears to prevent the secondary carbonyl‐aluminium interactions seen in ‘naked’ [(NON)AlFe(CO)_4_]^−^ (i. e. in **2‐crypt**) presumably by polarizing electron density in the iron carbonyl manifold. In the absence of the weak C→Al donor/acceptor interactions seen in **2‐crypt**, increased puckering of the NON xanthene framework in observed (angle between the two xanthene aromatic rings 122.5°, cf. 130.5° for **2‐crypt**), thereby allowing closer proximity of the xanthene O‐donor to Al1 (1.973(2) Å, cf. 2.029(1) Å for **2‐crypt**) to quench its higher Lewis acidity.

Quantum chemical analysis of **2‐crown** indicates that the major Fe−Al bonding orbital is the HOMO‐4; a WBI of 0.33 is calculated for the metal‐metal bond (*i. e*. similar to that for **2‐crypt**, 0.34), but no significant values are generated involving aluminium and any of the equatorial carbonyl ligand atoms. A Bader charge analysis reveal no significant differences in the charge distributions of the Fe(CO)_4_ (−1.62 e), (NON)Al (+0.68 e) or potassium‐containing fragments (+0.94 e) as compared to **2‐crypt**, although the iron centre itself possesses a lower partial positive charge (+0.09 e compared to +0.16 e), potentially due to the closer approach of the strongly sigma donating aluminyl ligand (crystallographically: 2.374(1) vs. 2.3938(7) Å).

**2‐crypt** and **2‐crown** represent the first examples of complexes featuring an aluminyl‐derived ligand bound to a binary iron carbonyl, with previously reported iron aluminyl systems relying on the availability of the nucleophilic iron centred anion CpFe(CO)_2_^−^. The possibility of filling this available chemical space through tuning of the aluminyl reactivity by counterion sequestration opens up a wide range of other potential transition metal substrates to which aluminyl ligands could be bound, with diverse onward chemistry. Such investigations are currently underway in our laboratory. However, preliminary experiments with the binary carbonyl Ni(CO)_4_, for example, yield only the reduced nickel carbonyl cluster [K(2.2.2‐cryptand)]_2_[Ni_8_(CO)_18_] (see ESI, Figure s1), indicating that this approach may not necessarily be uniformly generalizable to other 3*d* metal carbonyl species, given the very strongly reducing nature of the NON‐supported aluminyl ligand. In similar fashion, **2‐crown** is found to be unstable in solution, with prolonged storage of a THF solution yielding red single crystals of the potassium‐crown salt of known iron motif [K(18‐crown‐6]_2_[Fe_2_(CO)_8_], i. e. the formal product of the reductive coupling of two [Fe(CO)_4_] units; the fate of the aluminium‐containing component in this reaction is unclear.

### Syntheses and Structures of Gallyl‐ and Indyl‐Derived Complexes of Iron

**Scheme 2:**
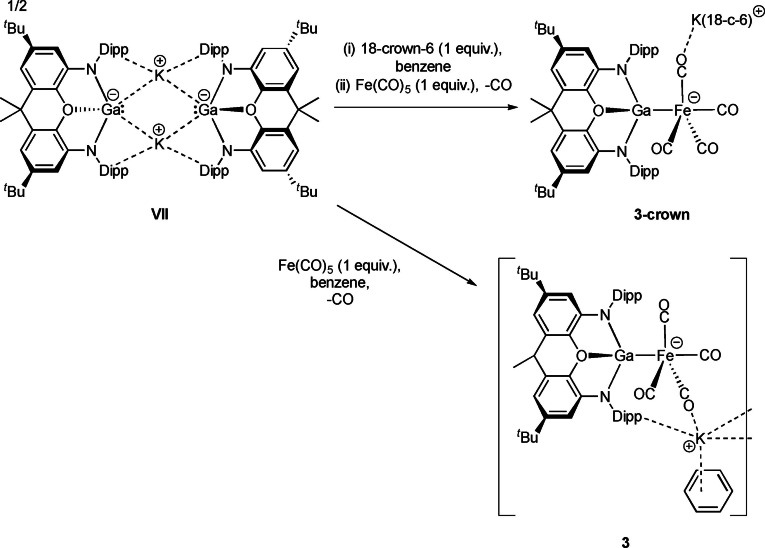
Synthesis of **3** and **3‐crown** from potassium gallyl dimer **VII**.

**Figure 6:**
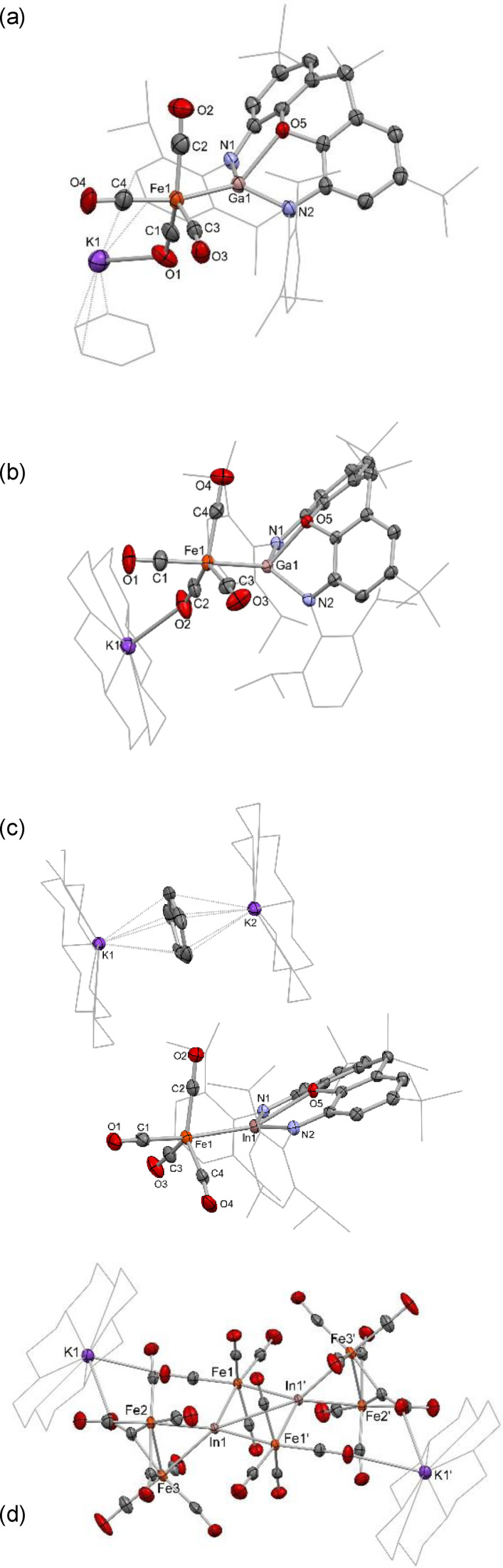
Molecular structures of (a) **3**, (b) **3‐crown**, (c) **4** and (d) **5**, as determined by X‐ray crystallography. H atoms and solvate molecules omitted, and some residues displayed in wireframe format for clarity. Key bond lengths (Å) and angles (°): (for **3**) Ga−Fe1 2.376(1), Ga1−O5 2.244(2), Ga1−N1 1.979(2), Ga1−N2 1.947(2), Fe1−C1 1.732(4), Fe1−C2 1.779(4), Fe1−C3 1.770(4), Fe1−C4 1.767(4), C1−O1 1.188(4), C2−O2 1.158(5), C3−O3 1.162(5), C4−O4 1.159(5), K1−O1 2.653(3), K1−O3 2.674(2), Ga−Fe1−C1 79.8(1), Ga1−Fe1−C2 78.1(1), Ga1−Fe1−C3 102.1(1), Ga−Fe1−C4 140.3(1), C1−Fe1−C2 134.3(2), C1−Fe1−C3 108.8(2), C2−Fe1−C3 114.7(2), C2−Fe1−C4 92.5(2); (for **3‐crown**): Ga1−Fe1 2.3518(7), Ga1−O5 2.125(2), Ga1−N1 1.951(3), Ga1−N2 1.970(3), Fe1−C1 1.763(4), Fe1−C2 1.751(4), Fe1−C3 1.778(3), Fe1−C4 1.773(4), C1−O1 1.163(5), C2−O2 1.172(5), C3−O3 1.149(5), C4−O4 1.159(5), K1−O2 2.677(3), Ga1−Fe1−C1 173.8(1), Ga1−Fe1−C2 81.1(1), C1−Fe1−C2 93.1(2), C2−Fe1−C3 124.5(2), C2−Fe1−C4 122.5(2), C3−Fe1−C4 110.2(2); (for **4**): In1−Fe1 2.5232(5), In1−O5 2.438(1), In1−N1 2.186(2), In1−N2 2.200(2), Fe1−C1 1.762(2), Fe1−C2 1.775(2), Fe1−C3 1.789(2), Fe1−C4 1.780(2), C1−O1 1.157(3), C2−O2 1.167(3), C3−O3 1.151(3), C4−O4 1.157(3), In1−Fe1−C1 165.51(8), In1−Fe1−C2 75.91(7), C1−Fe1−C2 95.6(1), C2−Fe1−C3 117.6(1), C2−Fe1−C4 131.3(1), C3−Fe1−C4 109.6(1).

With a convenient route to Fe−Al bonds in hand, we sought to extend these studies down group 13, in order to identify trends in the properties of the metallo‐ligands [(NON)E]^−^ as a function of E (E=Al, Ga, In). In contrast to the aluminium case, reaction of the potassium gallyl species K_2_[(NON)Ga]_2_ with Fe(CO)_5_ in the absence of any potassium sequestration agent directly yields the Fe−Ga bonded species [K(benzene)][(NON)GaFe(CO)_4_], **3**. The solid‐state structure of **3** determined crystallographically (Scheme 2 and Figure 6) reveals an Fe−Ga bond length (2.375(1) Å) which is very similar to the Fe−Al separation in **2‐crown** (2.374(1) Å), albeit as part of a coordination‐polymeric structure that brings about significant distortion of the coordination geometry at iron (∠Ga1−Fe1−C4=160.3(1)°; ∠Ga1−Fe1−C3=102.1(1)°; ∠Ga1−Fe1−C2=78.1(1)°). Potassium ions bridge adjacent [(NON)GaFe(CO)_4_]^−^ units via the Dipp group and O1 of one unit, and O3 and the other Dipp group of the second, and additionally show short contacts to a single molecule of benzene. The shortest Ga−C distance (Ga1−C2) however, is 2.657(3) Å, suggesting that carbonyl ligand interactions with the group 13 metal centre are not significant in this species, likely due to the lower Lewis acidity of gallium. Together with the lower magnitude of the reduction potential of gallium (and its reduced oxophilicity compared to aluminium), this likely underpins why no gallium isocarbonyl species (akin to **1**) is observed.^[6]^

Addition of 18‐crown‐6 to a solution of K_2_[(NON)Ga]_2_ prior to the addition of Fe(CO)_5_ leads to the formation of **3‐crown**, a Fe−Ga bonded species essentially isostructural with its aluminium congener (Scheme 2 and Figure 6). A similar TBP structure (τ_5_=0.82, ∠Ga1−Fe1−C1=171.4(1)°; ∠Ga1−Fe1−C2=81.1(1)°; ∠C1−Fe1−C2=93.1(2)°; sum of angles in equatorial plane, 357.2°) features a Fe−Ga bond of 2.3513(7) Å, which is both well within the sum of covalent radii (2.54 Å) and shorter than the Fe−Al bond in **2‐crown** (2.374(1) Å). The xanthene backbone is more planar than for **2‐crown**, allowing for a longer Ga−O bond (2.125(2) vs 1.973(2) Å), consistent with the more weakly Lewis acidic nature of the gallium metal centre.

DFT calculations (on the full contact ion pair) show that the HOMO‐4 is again the dominant metal‐metal bonding contribution, and the Fe−Ga bond returns a WBI of 0.44, i. e. larger than in either of the aluminium cases (0.33 and 0.34 for **2‐crown** and **2‐crypt**, respectively). A Bader charge analysis allocates significantly less negative charge to the Fe(CO)_4_ moiety as compared with the aluminium congener **3‐crown** (−0.99 vs. −1.62 e), and the (NON)Ga fragment is significantly less oxidised with an overall charge of +0.003 e. These parameters are consistent with the idea that the gallyl metallo‐ligand donates significantly less electron density into the iron carbonyl manifold. This decreased extent of electron release is also borne out by spectroscopic data (see below).

**Scheme 3:**
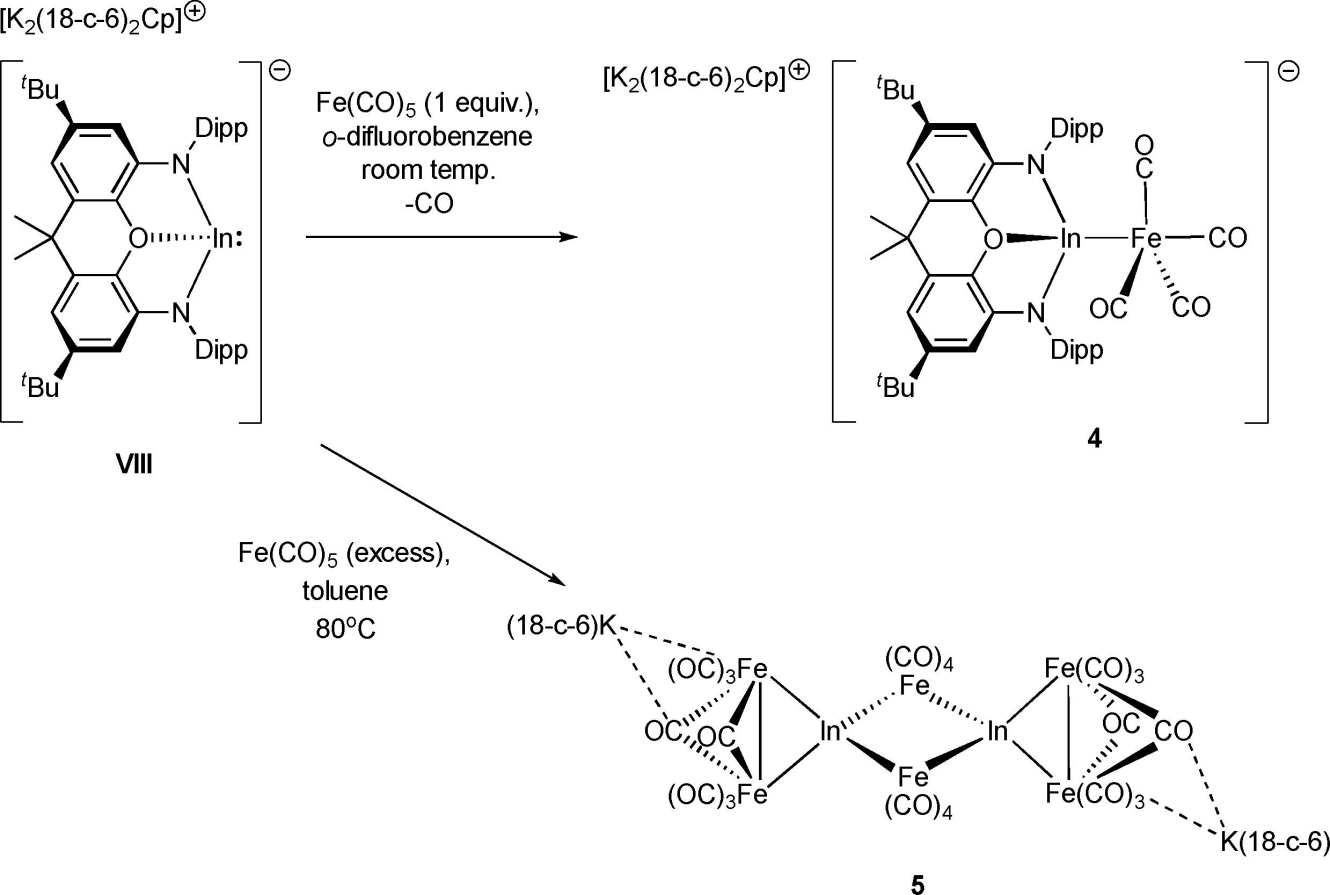
Syntheses of indyl complex **4** and mixed metal cluster **5** from indyl precursor **VIII**.

Moving finally to indyl‐derived systems, it was found necessary to use the crown‐sequestered separated‐ion‐pair form of the potassium indyl precursor, i. e. [K_2_(18‐crown‐6)_2_Cp][In(NON)] (**VIII**), for reasons of solubility.^[25]^ The Fe−In bonded product [K_2_(18‐crown‐6)_2_Cp][(NON)InFe(CO)_4_] (**4**) is formed in high yield when the reaction of **VIII** with Fe(CO)_5_ is carried out at room temperature in *ortho*‐difluorobenzene (Scheme 3). However, when the reaction is instead carried out in benzene, significant heating is required, which results in onward reaction with additional equivalents of Fe(CO)_5_ and subsequent isolation of large red crystals of the mixed‐metal carbonyl cluster **5** [K(18‐crown‐6)]_2_[(CO)_4_FeInFe_2_(CO)_8_]_2_. The fate of the NON ligand and the cation‐bound Cp are unknown.

Despite bearing no carbonyl‐potassium contacts due to K^+^ encapsulation within the [K_2_(18‐crown‐6)_2_Cp]^+^ cation, the indium‐iron fragment in **4** is otherwise isostructural with **2‐crown** and **3‐crown**, featuring an approximately TBP geometry at iron (τ_5_=0.57) with the group 13 metallo‐ligand binding in the axial position and no In−C close contacts present (∠In1−Fe1−C1=165.51(8)°; ∠In1−Fe1−C2=75.91(7)°; ∠C1−Fe1−C2 95.6(1)°; sum of angles in the equatorial plane, 358.8°). The metal‐metal linkage is again well within the sum of the respective covalent radii (*d*(In−Fe)=2.5232(5) Å, cf. 2.74 Å). Moreover, the larger (and more weakly Lewis acidic) indium centre leads to a significantly more planar xanthene backbone than for the lighter congeners, with the interplane angle between the two xanthene aromatic rings being 160.2°.

In line with **2‐crown** and **3‐crown**, the major Fe−In bonding orbital is again the HOMO‐4, and the WBI (0.47) is higher than for either the aluminium (0.33) or gallium congeners (0.44). Analysis of the Bader charges defines a trend down group 13, with a less electron rich [Fe(CO)_4_] manifold (−0.84 e) and a more electron rich (NON)In metallo‐ligand (−0.16 e). Consistent with diminished donor strength of the trielyl fragment down group 13, it seems that overall, the bonding is becoming less negatively polarised towards iron, despite the maximum in electronegativity at gallium.^[37]^ In order to probe these trends more fully, further computational analysis was undertaken by QT‐AIM methods. First, QT‐AIM analysis was carried out on the three trigonal‐bipyramidal adducts (Table 1). All three species feature a BCP between the two metals, with associated parameters which indicate metallic bonding in each case (low ρ, positive ∇^2^ρ) with very small differences between the three species.^[38]^ In each case the relatively low value of ρ at the BCP is consistent with the low Wiberg bond orders determined in each case. Within this group of complexes, the greater electron density at the BCP in the gallium case (**3‐crown**) indicates greater covalency, consistent with the higher electronegativity of gallium (over aluminium and indium) and with results previously reported for an isostructural series of compounds bearing Hg−E bonds (E=Al, Ga, In).^[25]^ The relative importance of ionic vs covalent bonding in the aluminyl‐derived compound **2‐crown** over its gallium counterpart **3‐crown** is reflected both in the lower WBI (0.33 vs 0.44) and in the more unsymmetrical charge distribution between the [Fe(CO)_4_] and E(NON) fragments (−1.62/+0.68 vs. −0.99/+0.003, for Fe/Al and Fe/Ga, respectively). Interestingly, this strongly polarized model of bonding for the aluminyl‐derived system resembles closely proposals made for the related system Cp*AlFe(CO)_4_.^[39]^ This aluminylene complex has been calculated to feature a (Mulliken) partial charge at aluminium very similar to that found in the unambiguous Al(III) system [Cp*_2_Al]^+^ (+0.70 vs. +0.71), and on this basis was described by Schnöckel and co‐workers as approaching a description [Cp*Al]^2+^[Fe(CO)_4_]^2−^ i.e. also closely related to Collman's reagent.

**Table 1:** Key QT‐AIM parameters for Fe−E Bond Critical Points (BCPs). A full table of parameters and contour plots is available in the ESI (Table s3; Figures s34–s37).

	**2‐crown** (Fe−Al)	**3‐crown** (Fe−Ga)	**4** (Fe−In)
ρ(r)/e Å^−3^	0.058	0.068	0.058
∇^2^ρ(r)/e Å^−5^	0.015	0.020	0.062

### Infrared and Mossbauer Spectroscopic Probes of Trielyl‐Derived [Fe(CO)_4_] Complexes

With examples of NON‐supported trielyl complexes of the type [(NON)EFe(CO)_4_]^−^ in hand, we wished to interrogate their electronic structures spectroscopically, making use of two convenient probes available to the [Fe(CO)_4_] fragment, namely IR and Mössbauer spectroscopies. The infrared spectra of **2‐crown**, **3‐crown** and **4** (Figures s9‐s13) each features four bands in the carbonyl region, with one (the *A*_1_ mode) well separated from the other three. These show the now established trend (**2‐crown**, 1967 cm^−1^; **3‐crown**, 1979 cm^−1^; **4**, 1981 cm^−1^), being found at higher energy/wavenumber for E=Ga and In (vs. Al), as a result of reduced back‐bonding from a less electron rich iron centre. This in turn reflects the more weakly σ‐donating nature of the gallyl and indyl ligands compared to their aluminyl counterpart.^[25]^ The difference in the position of the *A*_1_ band is significantly smaller between the gallyl and indyl complexes (compared to aluminyl/gallyl), which aligns with the more similar (and less polarized towards iron) charge distributions for these two complexes. This finding in turn is also consistent with the lower electronegativity differences between these elements (χ=1.61 (Al), 1.81 (Ga), 1.78 (In)). Comparison to other iron carbonyl complexes of comparable geometry is also possible. By this measure, all three complexes studied here yield an *A*_1_ mode at lower wavenumber than that of the isoelectronic carbene complex I^*i*Pr^Fe(CO)_4_ (2036 cm^−1^),^[40]^ as well as the neutral aluminylene complexes Cp*AlFe(CO)_4_ (2024 cm^−1^)^[41]^ and (Nacnac^Dipp^)AlFe(CO)_4_ (2007 cm^−1^),^[42]^ presumably due to the anionic nature of these trielyl systems. Comparing with other anionic systems, the isostructural complex of a diaza‐butadiene (DAB) supported gallyl anion is also known, which gives rise to an *A*_1_ stretching mode at 1988 cm^−1^.^[43]^ This indicates that the NON ligand framework yields a more strongly σ‐donating gallyl metallo‐ligand than the DAB framework, consistent with the results of previous studies of mercury trielyl compounds.^[25]^ Indeed, even the NON‐supported indyl anion exceeds the DAB‐supported gallyl in terms of donor strength by this metric.

**Figure 7:**
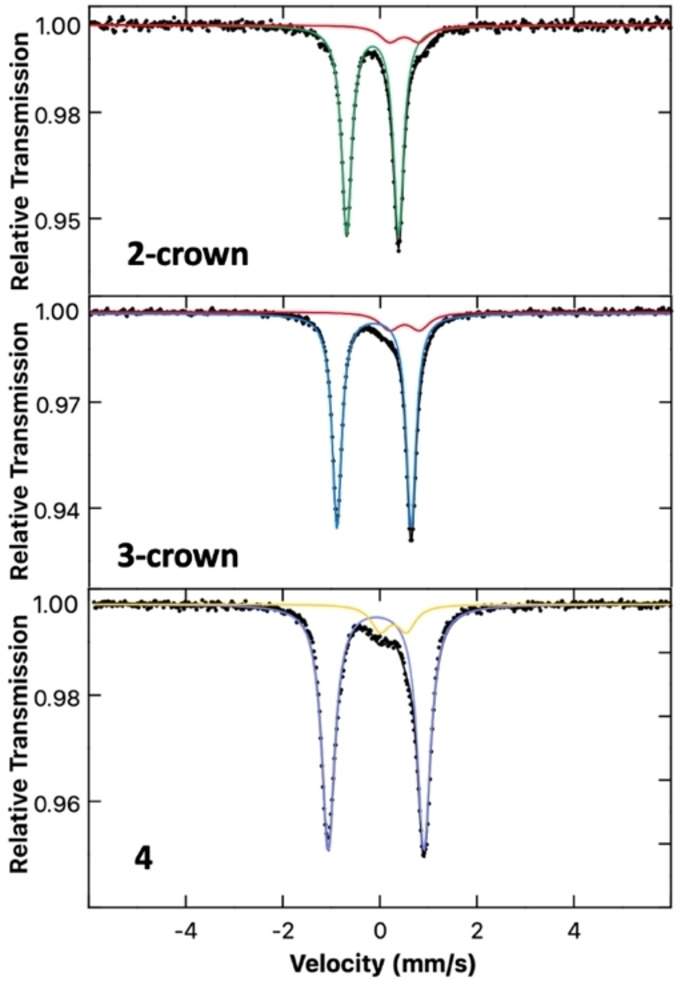
Zero field 80 K ^57^Fe Mössbauer spectrum of **2‐crown** (green, 88 %): δ=−0.24 mm/s, |ΔE_Q_|=2.08 mm/s, (red, 12 %): δ=0.50 mm/s, |ΔE_Q_|=0.61 mm/s; **3‐crown** (blue, 88 %): δ=−0.13 mm/s, |ΔE_Q_|=1.53 mm/s, (red, 12 %): δ=0.50 mm/s, |ΔE_Q_|=0.61 mm/s; **4** (purple, 88 %): δ=−0.08 mm/s, |ΔE_Q_|=1.97 mm/s, (yellow, 12 %): δ=0.28 mm/s, |ΔE_Q_|=0.54 mm/s.

For further comparison, the solid‐state Mössbauer spectrum was measured at 80 K for each of the three complexes (Figure 7). In the spectrum of **2‐crown**, the major species was identified as a doublet with parameters of δ=−0.24 mm/s and |ΔE_Q_|=2.08 mm/s with a minor component (red, δ=0.50 mm/s, |ΔE_Q_|=0.61 mm/s). The minor impurity can be identified as an oxidised iron‐carbonyl species, based on the similarity of the parameters to those reported for Fe(CO)_4_I_2_ (possibly resembling the cluster complex **5**).^[43]^ The major component of the spectrum of gallyl complex **3‐crown** is characterized by doublet parameters of δ=−0.13 mm/s and |ΔE_Q_|=1.53 mm/s. The parameters of **3‐crown** resemble those of **2‐crown** with a slightly more positive isomer shift and larger quadrupole splitting, as expected for a species in which a larger main group metal is coordinated to the Fe centre, resulting in a larger distortion of electronic distribution around the Fe centre.^[44]^ The minor impurity is the same in both complexes **2‐crown** and **3‐crown**. The Mössbauer parameters determined for complex **4** are δ=−0.08 mm/s and |ΔE_Q_|=1.97 mm/s; the minor species (yellow) is characterized by a signal at δ=0.28 mm/s and |ΔE_Q_|=0.54 mm/s, matching literature parameters for Fe_2_(CO)_9_.^[45]^

The parameters determined for complexes **2**‐crown, **3**‐crown and **4** follow a consistent trend, involving an increase of the isomer shift as the group is descended. The increase in the quadrupole splitting resulting from increased distortion in the electric field gradient is in good agreement with the idea of an increased electronic interaction in the case of larger group 13 elements. Few examples of Mössbauer studies can be found in literature involving iron(0) carbonyls bearing iron‐main group metal bonds, making these complexes novel in their characterization and providing valuable insight into the effect of trielyl coordination.

## Conclusions

We report an in‐depth investigation into the synthesis of trielyl‐derived Fe(CO)_4_ complexes, including a demonstration in the case of aluminyl complexes of how reactivity may be tuned by cation sequestration, ultimately to yield the desired metal‐metal bonded product. With the exception of **2‐crown**, the iron‐aluminium heterobimetallic species generated closely resemble analogues of Collman's reagent, in which one alkali metal cation has been formally been exchanged for [(NON)Al]^+^, bound in various modes. The synthesis of a series of isostructural complexes bearing direct bonds between iron and the aluminyl‐, gallyl‐ and indyl‐derived metallo‐ligands has enabled, in turn, a combined experimental and computational evaluation of metallo‐ligand properties. These reveal significant differences in electronic structure as the group is descended, most notably in terms of the polarisation of the Fe−E bond, which is highly negative at iron for the aluminyl‐derived species **2‐crown**, trending towards polarization in the opposite sense for indyl species **4**. These conclusions are supported by IR measurements based on the *A*_1_ stretching mode of the ancillary carbonyl ligand set. Mössbauer spectroscopy indicates an increased perturbation of the iron centred electric field gradient as group 13 is descended, consistent with interactions with increasingly large ligand donor atoms.

## Experimental

Included here are synthetic and characterizing data for compounds **1**, **2‐crown**, **3**, **3‐crown** and **4**. Complete data, representative spectra, and details of crystallographic^[46]^ and quantum chemical studies are included in the supporting information.

**1**: To a toluene (20 mL) solution of [K{Al(NON)}]_2_ (0.300 g, 0.41 mmol) was added Fe(CO)_5_ (0.067 mL, 0.5 mmol), at which point the solution immediately darkened. The solution was filtered into a lambda‐shaped J‐Youngs tube, which was stored under a partial internal vacuum with the sidearm cooled by a water bath overnight. Large, colourless single crystals were subsequently obtained from the concentrated supernatant solution. This solution was decanted, and the crystals washed with a minimum amount of cold hexane before drying *in vacuo*. Single crystal diffraction allowed identification of the crystals as K_2_[{(NON)Al}(OC)_2_Fe(CO)_2_]_2_ (**1**). Yield 0.148 g, 40 %. Elemental microanalysis: calc. for C_102_H_124_Al_2_Fe_2_K_2_N_4_O_10_: C 67.69 %, H 6.91 %, N 3.10 %; measured: 68.72 %, 7.16 %, 2.74 %. ^1^H NMR (500 MHz, THF‐d_8_, 298 K): δ_H_ 0.94 (d, ^3^ *J*_HH_ = 6.7 Hz, 6H, CH(C*H*_3_)_2_), 1.04 (d, ^3^ *J*_HH_ = 6.7 Hz, 6H, CH(C*H*_3_)_2_), 1.10 (d, ^3^ *J*_HH_ = 6.7 Hz, 6H, CH(C*H*_3_)_2_), 1.14 (s, 18H, C(*CH_3_*)_3_), 1.45 (d, ^3^ *J*_HH_ = 6.7 Hz, 6H, CH(C*H*_3_)_2_), 1.66 (s, 3H, C(C*H*_3_)_2_), 1.83 (s, 3H, C(C*H*_3_)_2_), 3.17 (sept, ^3^ *J*_HH_ = 6.7 Hz, 2H, C*H*(CH_3_)_2_), 3.87 (sept, ^3^ *J*_HH_ = 6.7 Hz, 2H, C*H*(CH_3_)_2_), 5.88 (d, ^4^ *J*_HH_ = 1.7 Hz, 2H, XA‐*o*‐C*H*), 6.68 (d, ^4^ *J*_HH_ = 1.7 Hz, 2H, XA‐*p*‐C*H*), 7.13–7.29 (m, 6H, Ar*H*). ^13^C{^1^H} NMR (101 MHz, THF‐d_8_, 298 K): δ_C_ 150.5, 148.0, 147.5, 143.8, 143.0, 138.7, 131.4, 126.9, 125.7, 124.6, 124.5, 111.2, 107.7, 37.6, 35.8, 34.3, 32.2, 29.1, 28.9, 27.4, 27.2, 24.8, 23.7.

**2‐crown**: To a Schlenk flask containing [K{Al(NON)}]_2_ (0.300 g, 0.41 mmol) and 18‐crown‐6 (0.108 g, 0.41 mmol) was added benzene (15 mL); brief sonication of the reaction mixture yielded an orange suspension. To this was added Fe(CO)_5_ (0.067 mL, 0.5 mmol), at which point the solution immediately darkened. Storage of the solution for one hour led to the formation of large colourless single crystals suitable for diffraction measurements. Decanting the supernatant and washing with the minimum volume of hexane before drying *in vacuo* allowed isolation of clean material of [K(18‐crown‐6)][(NON)AlFe(CO)_5_]. Yield 0.263 g, 55 %. Elemental microanalysis: calc for C_63_H_86_AlFeKN_2_O_11_: C 64.71 %, H 7.41 %, N 2.40 %; measured: C 64.80 %, H 7.52 %, N 2.08 %. ^1^H NMR (500 MHz, THF‐d_8_, 298 K): δ_H_ 0.80 (br. s, 6H, CH(C*H*_3_)_2_), 0.95 (d, ^3^ *J*_HH_ = 7.1 Hz, 6H, CH(C*H*_3_)_2_), 1.05 (d, ^3^ *J*_HH_ = 7.1 Hz, 6H, CH(C*H*_3_)_2_), 1.14 (s, 18H, C(*CH_3_*)_3_), 1.47 (d, ^3^ *J*_HH_ = 7.1 Hz, 6H, CH(C*H*_3_)_2_), 1.68 (s, 3H, C(C*H*_3_)_2_), 1.81 (s, 3H, C(C*H*_3_)_2_), 3.16 (sept, ^3^ *J*_HH_ = 6.6 Hz, 2H, C*H*(CH_3_)_2_), 3.61 (s, 24H, OC*H*_2_C*H*_2_O), 3.95 (sept, ^3^ *J*_HH_ = 6.6 Hz, 2H, C*H*(CH_3_)_2_), 5.86 (s, 1H, XA‐*o*‐C*H*), 5.90 (s, 1H, XA‐*o*‐C*H*), 6.50 (s, 1H, XA‐*p*‐C*H*), 6.65 (s, 1H, XA‐*p*‐C*H*), 7.00 (m, 4H, Ar‐*m*‐C*H*), 7.15 (m, 2H, Ar‐*p*‐C*H*). ^13^C{^1^H} NMR (101 MHz, THF‐d_8_, 298 K): δ_C_ 149.7, 147.1, 146.5, 146.3, 145.6, 144.0, 143.8, 142.9, 142.3, 128.0, 125.6, 124.5, 124.1, 123.1, 111.2, 109.9, 106.2, 105.3, 70.1, 34.6, 34.5, 31.1, 31.0, 28.0, 27.8, 26.1, 26.0, 22.5.

**3**: To a Schlenk flask containing [K{Ga(NON)}]_2_ (0.100 g, 0.128 mmol) and benzene (10 mL) was added Fe(CO)_5_ (0.018 mL, 0.13 mmol), at which point the solution immediately darkened. Storage of the solution for 12 h led to the formation of large needle‐shaped colourless single crystals suitable for diffraction measurements. Decanting the supernatant and washing with the minimum volume of hexane before drying *in vacuo* allowed isolation of clean K[(NON)GaFe(CO)_5_]. Yield 0.061 g, 50 %. Elemental micro‐analysis: calc. for C_51_H_62_FeGaKN_2_O_5_: C 66.74 %, H 6.68 %, N 2.73 %; measured: C 66.41 %, 6.74 %, 2.51 %. ^1^H NMR (500 MHz, THF‐d_8_, 298 K): δ_H_ 0.85 (br s, 12H, CH(C*H*_3_)_2_), 1.08 (s, 18H, C(*CH_3_*)_3_), 1.16 (m, 12H, CH(C*H*_3_)_2_), 1.71 (overlapping with residual solvent peak, 3H, C(C*H*_3_)_2_), 2.31 (s, 3H, C(C*H*_3_)_2_), 3.41 (br. m, 4H, C*H*(CH_3_)_2_), 5.85 (s, 2H, XA‐*o*‐C*H*), 6.44 (s, 2H, XA‐*p*‐C*H*), 7.00 (m, 4H, Ar‐*m*‐C*H*), 7.19 (m, 2H, Ar‐*p*‐C*H*). ^13^C{^1^H} NMR (101 MHz, THF‐d_8_, 298 K): δ_C_ 217.3, 143.8, 143.1, 142.6, 135.6, 134.3, 126.8, 126.1, 123.2, 122.3, 109.4, 103.0, 36.4, 32.5, 29.2, 26.1, 18.7.

**3‐crown**: To a Schlenk flask containing [K{Ga(NON)}]_2_ (0.200 g, 0.256 mmol) and 18‐crown‐6 (0.068 g, 0.256 mmol) was added benzene (20 mL) before brief sonication to yield a pale yellow suspension. To this was added Fe(CO)_5_ (0.036 mL, 0.26 mmol), at which point the solution immediately darkened slightly. Storage of the solution for one hour led to the formation of large colourless single crystals suitable for diffraction measurements. Decanting the supernatant and washing with the minimum volume of hexane before drying *in vacuo* allowed isolation of clean material of [K(18‐crown‐6)][(NON)GalFe(CO)_5_]. Yield 0.132 g, 43 %. Elemental microanalysis: calc. for C_63_H_86_FeGaKN_2_O_11_: C 63.49 %, H 5.58 %, N 2.35 %; measured: C 63.42 %, H 6.75 %, N 1.87 %.^1^H NMR (500 MHz, THF‐d_8_, 298 K): δ_H_ 0.85 (br s, 12H, CH(C*H*_3_)_2_), 1.08 (s, 18H, C(*CH_3_*)_3_), 1.19 (d, ^3^ *J*_HH_=6.9 Hz, 12H, CH(C*H*_3_)_2_), 1.67 (s, 3H, C(C*H*_3_)_2_), 2.31 (s, 3H, C(C*H*_3_)_2_), 3.38 (sept, ^3^ *J*_HH_=6.5 Hz, 4H, C*H*(CH_3_)_2_), 3.58 (s, 24H, OC*H*_2_C*H*_2_O), 5.86 (s, 2H, XA‐*o*‐C*H*), 6.43 (s, 2H, XA‐*p*‐C*H*), 7.08 (m, 2H, Ar‐*p*‐C*H*), 7.16 (m, 4H, Ar‐*m*‐C*H*). ^13^C{^1^H} NMR (101 MHz, THF‐d_8_, 298 K): δ_C_ 217.1, 146.1, 143.8, 143.3, 143.2, 142.7, 134.3, 126.8, 126.1, 123.2, 122.2, 121.6, 109.3, 102.8, 68.4, 36.4, 32.5, 29.3, 29.0, 26.0, 21.8, 21.6, 18.6.

**4**: To a Schlenk flask containing [K_2_(18‐crown‐6)_2_Cp][In(NON)] (0.2 g, 0.137 mmol) was added *o*‐difluorobenzene (20 mL), yielding a yellow suspension. To this was added Fe(CO)_5_ (0.019 mL, 0.14 mmol) before sonication at room temperature for 1 h, until the suspension clarified. Filtration and layering with hexane yielded large pale orange single crystals of (K_2_(18‐crown‐6)_2_Cp][(NON)InFe(CO)_4_] suitable for diffraction measurements. Yield 0.173 g, 87 %. Elemental microanalysis: calc. for C_80_H_115_FeInK_2_N_2_O_17_: C 59.37 %, H 6.89 %, N 1.61 %. Measured: C 59.20 %, H 6.92 %, N 1.34 %. ^1^H NMR (500 MHz, benzene‐d_6_, 298 K): δ_H_ 1.34 (d, ^3^ *J*_HH_ = 6.7 Hz, 12H, CH(C*H*_3_)_2_), 1.35 (s, 18H, C(*CH_3_*)_3_), 1.71 (d, ^3^ *J*_HH_ = 6.7 Hz, 12H, CH(C*H*_3_)_2_), 1.79 (s, 6H, C(C*H*_3_)_2_), 3.20 (s, 48H, OC*H*_2_C*H*_2_O), 4.09 (sept, 4H, C*H*(CH_3_)_2_), 6.14 (s, 5H, C_5_ *H*_5_), 6.41 (d, ^4^ *J*_HH_ = 2.1 Hz, 2H, XA‐o‐C*H*), 6.75 (d, ^4^ *J*_HH_ = 2.1 Hz, 2H, XA‐*p*‐C*H*), 7.39 (m, 2H, Ar‐*p*‐C*H*), 7.48 (d, 4H, Ar‐*m*‐C*H*). ^13^C{^1^H} NMR (101 MHz, benzene‐d_6_, 298 K): δ_C_ 219.0, 147.9, 146.2, 145.2, 142.9, 134.7, 125.3, 124.2, 110.4, 104.9, 104.4, 37.3, 35.1, 32.2, 28.8, 27.9, 26.2, 25.2.

## Supporting Information

The authors have cited additional references within the Supporting Information (Ref. [47–61]).

## Conflict of Interests

The authors declare no conflict of interest.

1

## Supporting information

As a service to our authors and readers, this journal provides supporting information supplied by the authors. Such materials are peer reviewed and may be re‐organized for online delivery, but are not copy‐edited or typeset. Technical support issues arising from supporting information (other than missing files) should be addressed to the authors.

Supporting Information

## Data Availability

The data that support the findings of this study are available in the supplementary material of this article.
