# Supplementary material for: Synthesis, and Structural and Spectroscopic Analysis of Trielyl‐Derived Complexes of Iron
Source: Chemistry. 2025 Mar 5;31(18):e202404451. doi: 10.1002/chem.202404451 (PMC11937870; doi:10.1002/chem.202404451)
Supplement: Supplementary file 1 — Supporting Information [file CHEM-31-e202404451-s001.pdf]

Chemistry–A European Journal

Supporting Information

Synthesis, and Structural and Spectroscopic Analysis of Trielyl-Derived Complexes of Iron

Liam P. Griffin, Alexis K. Bauer, Agamemnon E. Crumpton, Mathias A. Ellwanger, Andreas Heilmann, Anja Wiesner, Michael L. Neidig, and Simon Aldridge*

Synthesis, and Structural and Spectroscopic Analysis of Iron-Trielyl Complexes

Liam P. Griffin, Alexis K. Bauer, Agamemnon E. Crumpton, Mathias A. Ellwanger, Andreas Heilmann, Anja Wiesner, Michael L. Neidig and Simon Aldridge

Inorganic Chemistry Laboratory, Department of Chemistry, University of Oxford, South Parks Road, Oxford, OX1 3QR (UK), and

Institute of Inorganic Chemistry, Freie Universität Berlin, Fabeckstr. 34/36, 14195 Berlin (Germany)

Supporting information

1. General considerations s2
2. X-ray crystallographic details s3
3. Syntheses of novel compounds s6
4. NMR spectra of novel compounds s10
5. IR spectra of novel compounds s16
6. Mössbauer spectra of novel compounds s19
7. Computational details s22
8. xyz Coordinates of calculated structures s38
9. References for supporting information s49

1. General considerations

All manipulations were carried out using standard Schlenk line or dry-box techniques under an atmosphere of argon or dinitrogen. Solvents were degassed by sparging with argon and dried by passing through a column of the appropriate drying agent. Toluene was purified using an MBraun SPS-800 and stored over a potassium mirror. *o*-difluorobenzene was dried by storage over molecular sieves before degassing by three freeze pump thaw cycles. NMR spectra were measured in benzene-d_6 (which was dried over potassium, with the solvent then being distilled under reduced pressure), or THF-d_8 (dried by storing over activated molecular sieves and degassed by three freeze pump thaw cycles). NMR samples were prepared under argon in 5 mm Wilmad 507-PP tubes fitted with J. Young Teflon valves. ^{1}H and $^{13}C\{^{1}H\}$ NMR spectra were recorded on Bruker Avance III HD nanobay 400 MHz or Bruker Avance III 500 MHz spectrometer at ambient temperature and referenced internally to residual protio-solvent (^{1}H) or solvent (^{13}C) resonances and are reported relative to tetramethylsilane (δ = 0 ppm). Assignments were confirmed using two-dimensional ^{1}H-^{1}H and ^{13}C-^{1}H NMR correlation experiments. Chemical shifts are quoted in δ (ppm) and coupling constants in Hz. Elemental analyses were carried out by London Metropolitan University or Elemental Microanalysis Ltd. $Fe(CO)_5$ was stored over molecular sieves in a freezer in a glovebox and thawed immediately before each use. [2.2.2.]-cryptand was purified by sublimation under vacuum. 18-crown-6 was recrystallised from hot acetonitrile and dried under vacuum. $Ni(CO)_4$ was used as received. $[K\{Al(NON)\}]_2$, $[K\{Ga(NON)\}]_2$, and $[K_2(18\text{-crown-}6)_2Cp][In(NON)]$ were prepared according to literature procedures.[s1, s2]

2. X-ray crystallographic details

Single-crystal X-ray diffraction data for compounds **1**, **2-crypt**, **2-crown**, **3**, **3-crown**, **4**, **5** and **6** were collected on an Oxford Diffraction/Agilent SuperNova diffractometer equipped with a 135 mm Atlas CCD area detector or a Rigaku XtaLAB Synergy-DW VHF equip with a PhotonJet-R dual wavelength rotating anode and HyPix-Arc 150° detector. Crystals were selected under Paratone-N oil, mounted on MiTeGen Micromount loops and quench-cooled using an Oxford Cryosystems open flow N2 cooling device.[s3] Data were collected at 100 or 150 K using mirror monochromated Cu Kα radiation (λ = 1.5418 Å; Oxford Diffraction Supernova) or Mo Kα radiation (λ = 0.71073 Å; Oxford Diffraction Supernova). Data collected were processed using the CrysAlisPro package, including unit cell parameter refinement and inter-frame scaling (which was carried out using SCALE3 ABSPACK within CrysAlisPro).[s4] Equivalent reflections were merged and diffraction patterns processed with the CrysAlisPro suite.[s4] Structures were solved ab initio from the integrated intensities using SHELXT[s5] and refined on F2 using SHELXL[s6] with the graphical interface OLEX2.[s7] Selected crystallographic data are summarised in Table s1.

Table s1: X-ray crystallographic details

	1	2-crypt	2-crown	3	3-crown	4	5	6
**Formula**	$C_{102}H_{124}Al_2Fe_2K_2N_4O_{10}$, 4($C_7H_8$)	$C_{87}H_{116}AlFeKN_4O_{11}$	$C_{63}H_{86}AlFeKN_2O_{11}$, 4($C_6H_6$)	$C_{57}H_{68}FeGaKN_2O_5$, 2.5(C_6H_6)	$C_{63}H_{86}FeGaKN_2O_{11}$, 1.5($C_6H_6$)	$C_{51}H_{62}FeInN_2O_5$, $C_{35}H_{59}K_2O_{12}$, 0.5(C_6H_6), 0.5(C_6H_{14})	$C_{48}H_{48}Fe_6In_2K_2O_{36}$	$C_{54}H_{72}K_2N_4Ni_8O_{30}$
**Fw (g mol^{-1})**	2178.44	1515.76	1481.69	1221.07	1329.16	1785.85	1843.80	1805.03
**Crystal system**	Monoclinic	Triclinic	Monoclinic	Monoclinic	Triclinic	Monoclinic	Triclinic	Triclinic
**Space group**	I 1 2/a 1	P -1	P 1 c 1	I 1 2/a 1	P -1	P 1 2_1/c 1	P -1	P -1
**a (Å)**	24.2358(5)	13.0235(2)	25.0904(3)	21.9032(2)	12.7340(2)	12.63069(4)	9.5811(3)	11.2851(10)
**b (Å)**	18.6924(4)	13.8732(2)	12.4623(2)	13.86540(10)	23.0469(3)	31.79286(9)	13.0624(3)	13.3656(14)
**c (Å)**	26.7388(7)	23.1100(4)	26.4474(3)	43.2338(4)	24.7089(4)	23.60319(7)	14.1928(3)	24.497(2)
**α (°)**	90	95.0460(10)	90	90	89.0350(10)	90	87.307(2)	82.483(7)
**β (°)**	104.412(3)	93.8940(10)	96.0360(10)	90.3340(10)	78.4690(10)	98.2952(3)	89.670(2)	87.949(7)
**γ (°)**	90	91.1300(10)	90	90	89.3030(10)	90	72.843(2)	79.684(7)
**V (Å^{-3})**	11732.2(5)	4148.29(11)	8223.83(19)	13129.7(2)	7103.90(19)	9379.07(5)	1695.28(8)	3603.6(6)
**Z**	4	2	4	8	4	4	1	2
**ρ_{calc} (g cm^{-3})**	1.233	1.214	1.197	1.235	1.243	1.265	1.806	1.664
**Radiation, λ (Å)**	1.54184	1.54184	1.54184	1.54184	1.54184	1.54184	1.54184	1.54184
**Absorption**	Multi-scan	Gaussian	Gaussian	Gaussian	Analytical	Multi-scan	Multi-scan	Multi-scan
**μ (mm^{-1})**	3.231	2.486	2.490	3.228	3.086	4.507	17.230	3.973
**Reflections collected**	86175	65512	84967	94154	42712	257953	33594	63026
**Independent reflections**	12214	17106	27505	13376	42712	19375	7010	13003
**$R_{(int)}$**	0.0681	0.0362	0.0532	0.0626	N/A, Twin integration performed	0.0356	0.0492	0.1293
**Parameters**	694	993	1895	863	1741	1071	587	1353
**R_1 (all data/I>2σ(I))**	0.0642	0.0505	0.0409	0.0519	0.0482	0.0346	0.0291	0.0892
**ωR_2 (all data/I >2σ(I))**	0.1830	0.1455	0.0979	0.1385	0.1290	0.0906	0.0771	0.2294
**GooF**	1.042	1.037	1.016	1.049	0.940	1.071	1.029	1.029
**T (K)**	150.00(10)	150.01(10)	150.00(10)	100.00(10)	150.01(10)	100.00(10)	150.00(10)	150.00
**CCDC ref**	2404083	2404085	2404087	2404088	2404086	2404089	2404082	2404084

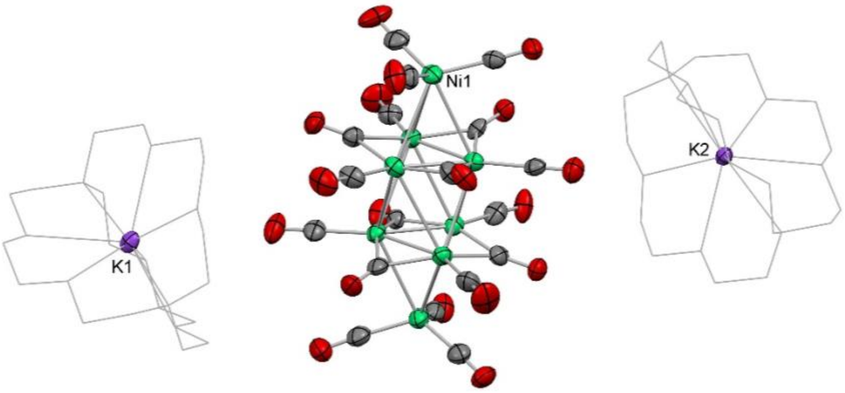

Figure s1: X-ray structure of $[K(2.2.2\text{-cryptand})]_2[Ni_8(CO)_{18}]$, **6**

3. Syntheses of novel compounds

1: To a toluene (20 mL) solution of $[K\{Al(NON)\}]_2$ (300 mg, 0.41 mmol) was added $Fe(CO)_5$ (0.067 mL, 0.5 mmol), at which point the solution immediately darkened. The solution was filtered into a lambda-shaped J-Youngs tube, which was stored under a partial internal vacuum with the sidearm cooled by a water bath overnight. Large, colourless single crystals were subsequently obtained from the concentrated supernatant solution. This solution was decanted, and the crystals washed with a minimum amount of cold hexane before drying *in vacuo*. Single crystal diffraction allowed identification of the crystals as $K_2[\{(NON)Al\}(OC)_2Fe(CO)_2]_2$ (**1**). Yield 148 mg, 40%.

Calc. for $C_{102}H_{124}Al_2Fe_2K_2N_4O_{10}$: C 67.69%, H 6.91%, N 3.10%. Measured: 68.72%, 7.16%, 2.74%

^1H NMR (500 MHz, THF-d_8, 298 K): δ_H 0.94 (d, $^3J_{HH}$ = 6.7 Hz, 6H, $CH(CH_3)_2$), 1.04 (d, $^3J_{HH}$ = 6.7 Hz, 6H, $CH(CH_3)_2$), 1.10 (d, $^3J_{HH}$ = 6.7 Hz, 6H, $CH(CH_3)_2$), 1.14 (s, 18H, $C(CH_3)_3$), 1.45 (d, $^3J_{HH}$ = 6.7 Hz, 6H, $CH(CH_3)_2$), 1.66 (s, 3H, $C(CH_3)_2$), 1.83 (s, 3H, $C(CH_3)_2$), 3.17 (sept, $^3J_{HH}$ = 6.7 Hz, 2H, $CH(CH_3)_2$), 3.87 (sept, $^3J_{HH}$ = 6.7 Hz, 2H, $CH(CH_3)_2$), 5.88 (d, $^4J_{HH}$ = 1.7 Hz, 2H, XA-*o*-C*H*), 6.68 (d, $^4J_{HH}$ = 1.7 Hz, 2H, XA-*p*-C*H*), and 7.13-7.29 (m, 6H, Ar*H*) ppm.

$^{13}C\{^1H\}$ NMR (101 MHz, THF-d_8, 298 K): δ_C 150.5, 148.0, 147.5, 143.8, 143.0, 138.7, 131.4, 126.9, 125.7, 124.6, 124.5, 111.2, 107.7, 37.6, 35.8, 34.3, 32.2, 29.1, 28.9, 27.4, 27.2, 24.8 and 23.7 ppm.

2-cryptand: To an NMR tube fitted with a J-Young valve containing $[K\{Al(NON)\}]_2$ (0.02g, 0.027 mmol) and [2.2.2]-cryptand (0.01 g, 0.02 mmol) was added benzene (0.3 mL) before brief sonication to yield a red suspension. To this was added $Fe(CO)_5$ (0.04 mL 0.030 mmol), at which point the solution immediately darkened. Storage of this solution for 1 week yielded a small quantity of pale yellow crystals suitable for diffraction measurements, which identified the product as [K(2.2.2-cryptand)][(NON)AlFe$(CO)_4$]. Attempts to crystallize the material by other methods or at larger scale did not significantly increase the yield of material beyond the small amounts of crystals isolated by this procedure. These crystals were found to be highly insoluble in compatible solvents, so only *in situ* NMR data of the non-selective reaction could be collected.

2-crown: To a Schlenk flask containing $[K\{Al(NON)\}]_2$ (0.300 g, 0.41 mmol) and 18-crown-6 (0.108 g, 0.41 mmol) was added benzene (15 mL) before brief sonication to yield an orange suspension. To this was added $Fe(CO)_5$ (0.067 mL, 0.5 mmol), at which point the solution immediately darkened. Storage of the solution for one hour led to the formation of large colourless single crystals suitable for

diffraction measurements. Decanting the supernatant and washing with the minimum volume of hexane before drying *in vacuo* allowed isolation of clean material of [K(18-crown-6)][(NON)AlFe(CO)$_5$]. Yield 0.263 g, 55%.

Calc for $C_{63}H_{86}AlFeKN_2O_{11}$: C 64.71%, H 7.41%, N 2.40%. Found: C 64.80%, H 7.52%, N 2.08%

^{1}H NMR (500 MHz, THF-d$_8$, 298 K): δ_H 0.80 (br. s, 6H, CH(C*H*$_3$)$_2$), 0.95 (d, $^3J_{HH}$ = 7.1 Hz, 6H, CH(C*H*$_3$)$_2$), 1.05 (d, $^3J_{HH}$ = 7.1 Hz, 6H, CH(C*H*$_3$)$_2$), 1.14 (s, 18H, C(C*H*$_3$)$_3$), 1.47 (d, $^3J_{HH}$ = 7.1 Hz, 6H, CH(C*H*$_3$)$_2$), 1.68 (s, 3H, C(C*H*$_3$)$_2$), 1.81 (s, 3H, C(C*H*$_3$)$_2$), 3.16 (sept, $^3J_{HH}$ = 6.6 Hz, 2H, C*H*(CH$_3$)$_2$), 3.61 (s, 24H, OC*H*$_2$C*H*$_2$O), 3.95 (sept, $^3J_{HH}$ = 6.6 Hz, 2H, C*H*(CH$_3$)$_2$), 5.86 (s, 1H, XA-*o*-C*H*), 5.90 (s, 1H, XA-*o*-C*H*), 6.50 (s, 1H, XA-*p*-C*H*), 6.65 (s, 1H, XA-*p*-C*H*), 7.00 (m, 4H, Ar-*m*-C*H*) and 7.15 (m, 2H, Ar-*p*-C*H*) ppm.

^{13}C{^{1}H} NMR (101 MHz, THF-d$_8$, 298 K): δ_C 149.7, 147.1, 146.5, 146.3, 145.6, 144.0, 143.8, 142.9, 142.3, 128.0, 125.6, 124.5, 124.1, 123.1, 111.2, 109.9, 106.2, 105.3, 70.1, 34.6, 34.5, 31.1, 31.0, 28.0, 27.8, 26.1, 26.0 and 22.5 ppm.

3: To a Schlenk flask containing [K{Ga(NON)}]$_2$ (0.1 g, 0.128 mmol) and benzene (10 mL) was added Fe(CO)$_5$ (0.018 mL, 0.13 mmol), at which point the solution immediately darkened. Storage of the solution for 12 hours led to the formation of large needle-shaped colourless single crystals suitable for diffraction measurements. Decanting the supernatant and washing with the minimum volume of hexane before drying *in vacuo* allowed isolation of clean material of K[(NON)GaFe(CO)$_5$]. Yield 0.061g, 50%.

Calc. for $C_{51}H_{62}FeGaKN_2O_5$: C 66.74%, H 6.68%, N 2.73%. Measured: C 66.41%, 6.74%, 2.51%

^{1}H NMR (500 MHz, THF-d$_8$, 298 K): δ_H 0.85 (br. s, 12H, CH(C*H*$_3$)$_2$), 1.08 (s, 18H, C(C*H*$_3$)$_3$), 1.16 (m, 12H, CH(C*H*$_3$)$_2$), 1.71 (overlapping with residual solvent peak, 3H, C(C*H*$_3$)$_2$), 2.31 (s, 3H, C(C*H*$_3$)$_2$), 3.41 (br. m, 4H, C*H*(CH$_3$)$_2$), 5.85 (s, 2H, XA-*o*-C*H*), 6.44 (s, 2H, XA-*p*-C*H*), 7.00 (m, 4H, Ar-*m*-C*H*) and 7.19 (m, 2H, Ar-*p*-C*H*) ppm.

^{13}C{^{1}H} NMR (101 MHz, THF-d$_8$, 298 K): δ_C 217.3, 143.8, 143.1, 142.6, 135.6, 134.3, 126.8, 126.1, 123.2, 122.3, 109.4, 103.0, 36.4, 32.5, 29.2, 26.1 and 18.7 ppm.

3-crown: To a Schlenk flask containing [K{Ga(NON)}]$_2$ (0.2 g, 0.256 mmol) and 18-crown-6 (0.068 g, 0.256 mmol) was added benzene (20 mL) before brief sonication to yield a pale yellow suspension. To this was added Fe(CO)$_5$ (0.036 mL, 0.26 mmol), at which point the solution immediately darkened slightly. Storage of the solution for one hour led to the formation of large colourless single crystals

suitable for diffraction measurements. Decanting the supernatant and washing with the minimum volume of hexane before drying *in vacuo* allowed isolation of clean material of [K(18-crown-6)][(NON)GalFe(CO)$_5$]. Yield 0.132g, 43%

Calc. for $C_{63}H_{86}FeGaKN_2O_{11}$: C 63.49%, H 5.58%, N 2.35%. Measured: C 63.42%, H 6.75%, N 1.87%

^{1}H NMR (500 MHz, THF-d$_8$, 298 K): δ_H 0.85 (br. s, 12H, CH(C*H*$_3$)$_2$), 1.08 (s, 18H, C(*CH$_3$*)$_3$), 1.19 (d, $^3J_{HH}$ = 6.9 Hz, 12H, CH(C*H*$_3$)$_2$), 1.67 (s, 3H, C(C*H*$_3$)$_2$), 2.31 (s, 3H, C(C*H*$_3$)$_2$), 3.38 (sept, $^3J_{HH}$ = 6.5 Hz, 4H, C*H*(CH$_3$)$_2$), 3.58 (s, 24H, OC*H*$_2$C*H*$_2$O), 5.86 (s, 2H, XA-*o*-C*H*), 6.43 (s, 2H, XA-*p*-C*H*), 7.08 (m, 2H, Ar-*p*-C*H*) and 7.16 (m, 4H, Ar-*m*-C*H*) ppm.

^{13}C{^{1}H} NMR (101 MHz, THF-d$_8$, 298 K): δ_C 217.1, 146.1, 143.8, 143.3, 143.2, 142.7, 134.3, 126.8, 126.1, 123.2, 122.2, 121.6, 109.3, 102.8, 68.4, 36.4, 32.5, 29.3, 29.0, 26.0, 21.8, 21.6 and 18.6 ppm.

4: To a Schlenk flask containing $[K_2(18\text{-crown-}6)_2Cp][In(NON)]$ (0.2 g, 0.137 mmol) was added *o*-DFB (20 mL), yielding a yellow suspension. To this was added $Fe(CO)_5$ (0.019 mL, 0.14 mmol) before sonication at room temperature for 1 h, until the suspension clarified. Filtration and layering with hexane yielded large pale orange single crystals of $(K_2(18\text{-crown-}6)_2Cp][(NON)InFe(CO)_4]$ suitable for diffraction measurements. Yield 0.173 g, 87%.

Calc. for $C_{80}H_{115}FeInK_2N_2O_{17}$: C 59.37%, H 6.89%, N 1.61%. Measured: C 59.20%, H 6.92%, N 1.34%

^{1}H NMR (500 MHz, benzene-d$_6$, 298 K): δ_H 1.34 (d, $^3J_{HH}$ = 6.7 Hz, 12H, CH(C*H*$_3$)$_2$), 1.35 (s, 18H, C(*CH$_3$*)$_3$), 1.71 (d, $^3J_{HH}$ = 6.7 Hz, 12H, CH(C*H*$_3$)$_2$), 1.79 (s, 6H, C(C*H*$_3$)$_2$), 3.20 (s, 48H, OC*H*$_2$C*H*$_2$O), 4.09 (sept., 4H, C*H*(CH$_3$)$_2$), 6.14 (s, 5H, C$_5$*H*$_5$), 6.41 (d, $^4J_{HH}$ = 2.1 Hz, 2H, XA-o-C*H*), 6.75 (d, $^4J_{HH}$ = 2.1 Hz, 2H, XA-*p*-C*H*), 7.39 (m, 2H, Ar-*p*-C*H*) and 7.48 (d, 4H, Ar-*m*-C*H*) ppm.

^{13}C{^{1}H} NMR (101 MHz, benzene-d$_6$, 298 K): δ_C 219.0, 147.2, 146.2, 145.2, 142.9, 134.7, 125.3, 124.2, 110.4, 104.9, 104.4, 37.3, 35.1, 32.2, 28.8, 27.9, 26.2 and 25.2 ppm.

5: To a Schlenk flask containing $[K_2(18\text{-crown-}6)_2Cp][In(NON)]$ (0.1 g, 0.067 mmol) was added benzene (10 mL), yielding a yellow suspension. To this was added $Fe(CO)_5$ (0.012 mL, 0.1 mmol) before heating to 80 °C for 2 h. Filtration and layering with hexane yielded a mixture of crystals of **4** and the mixed metal cluster compound **5**, which was characterised crystallographically. The fate of the NON containing component is unknown.

$[K(2.2.2\text{-cryptand})]_2[Ni_8(CO)_{18}]$, 6: To a J-Young flask containing $[K\{Al(NON)\}]_2$ (0.02g, 0.027 mmol) and [2.2.2]-cryptand (0.01 g, 0.027 mmol) was added benzene (0.3 mL) before brief sonication and degassing with three freeze pump thaw cycles. A small excess (as judged by weighing of the tube) of $Ni(CO)_4$ was then vacuum transferred onto the frozen solution before allowing it to thaw. The solution turned dark red, and storage for 6 h yielded small quantities of red crystals of **6**, which was characterised crystallographically. The fate of the aluminium containing component is unknown.

4. NMR spectra of novel compounds

Compound 1

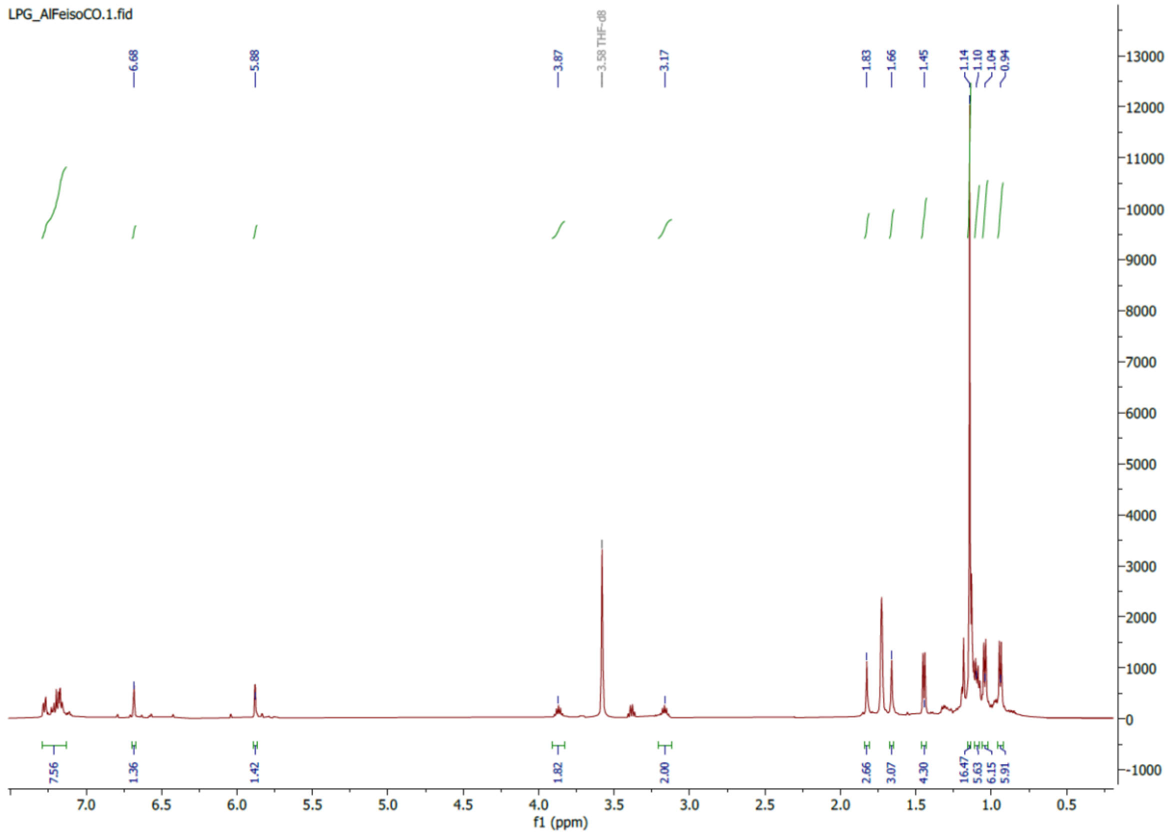

Figure s2: ^{1}H NMR spectrum of **1**

Compound 2-crypt

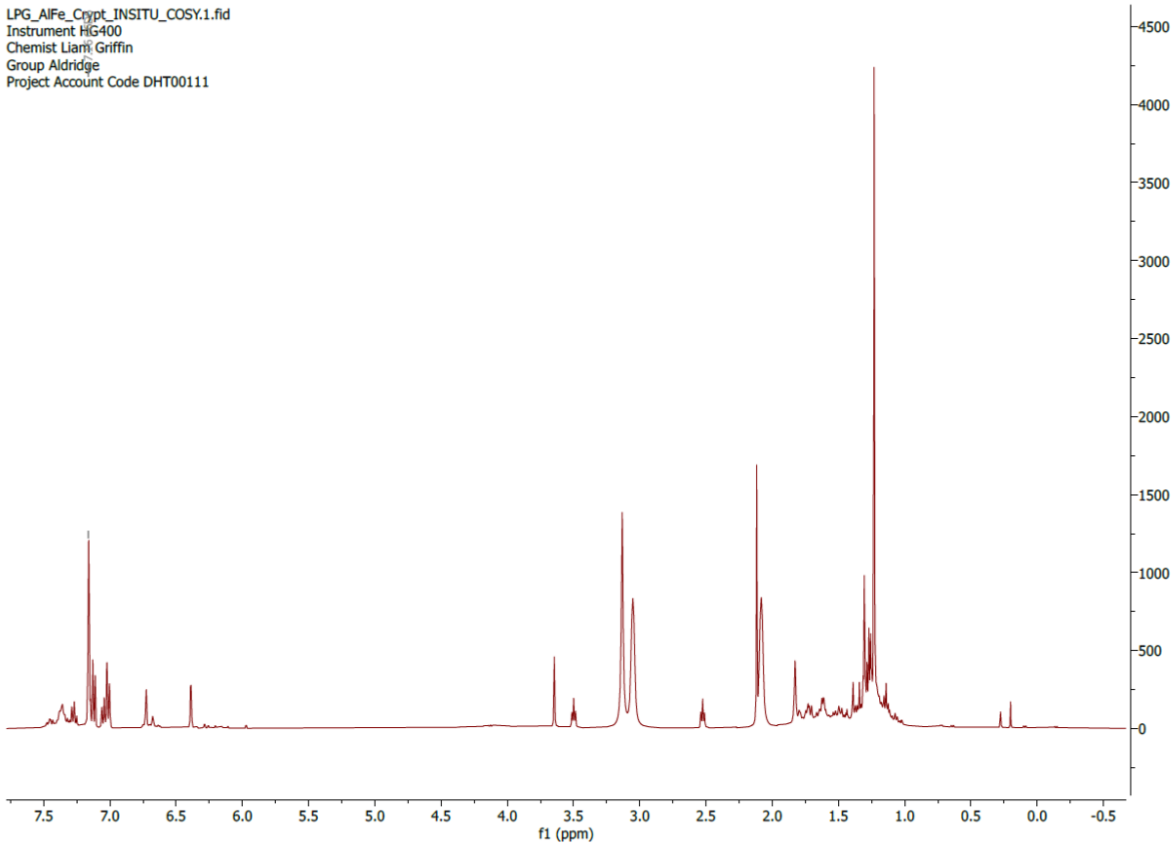

Figure s3: *In situ* ^{1}H NMR spectrum of **2-crypt**, which indicates that an unselective reaction has occurred

Compound 2-crown

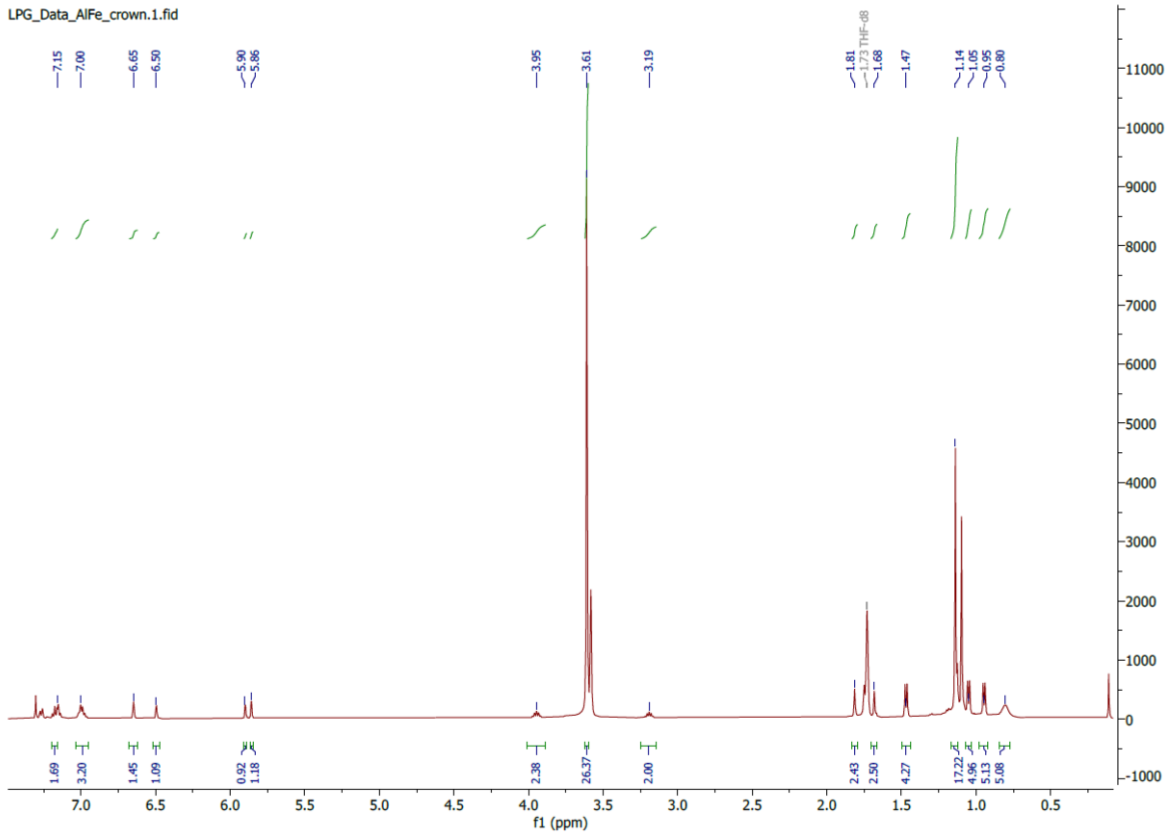

Figure s4: ^{1}H NMR spectrum of **2-crown**

Compound 3

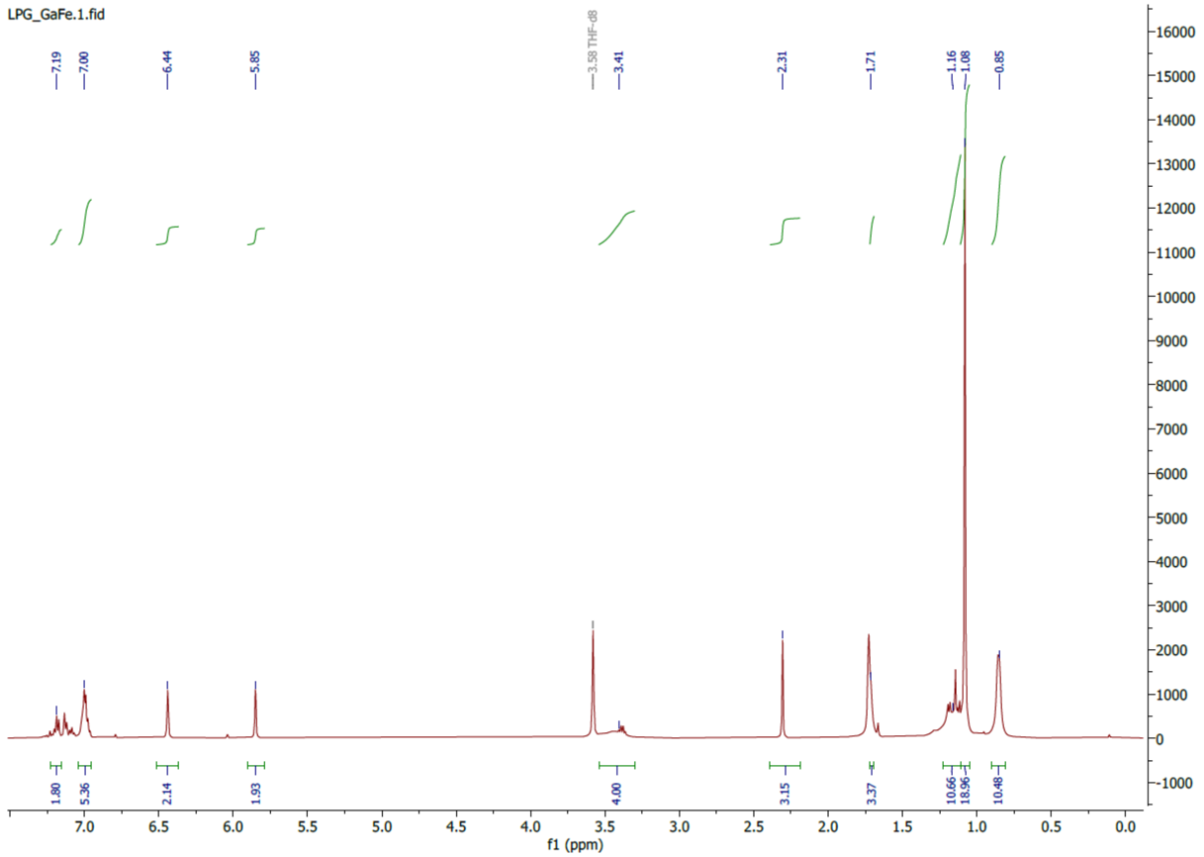

Figure s5: ^{1}H NMR spectrum of **3**

Compound 3-crown

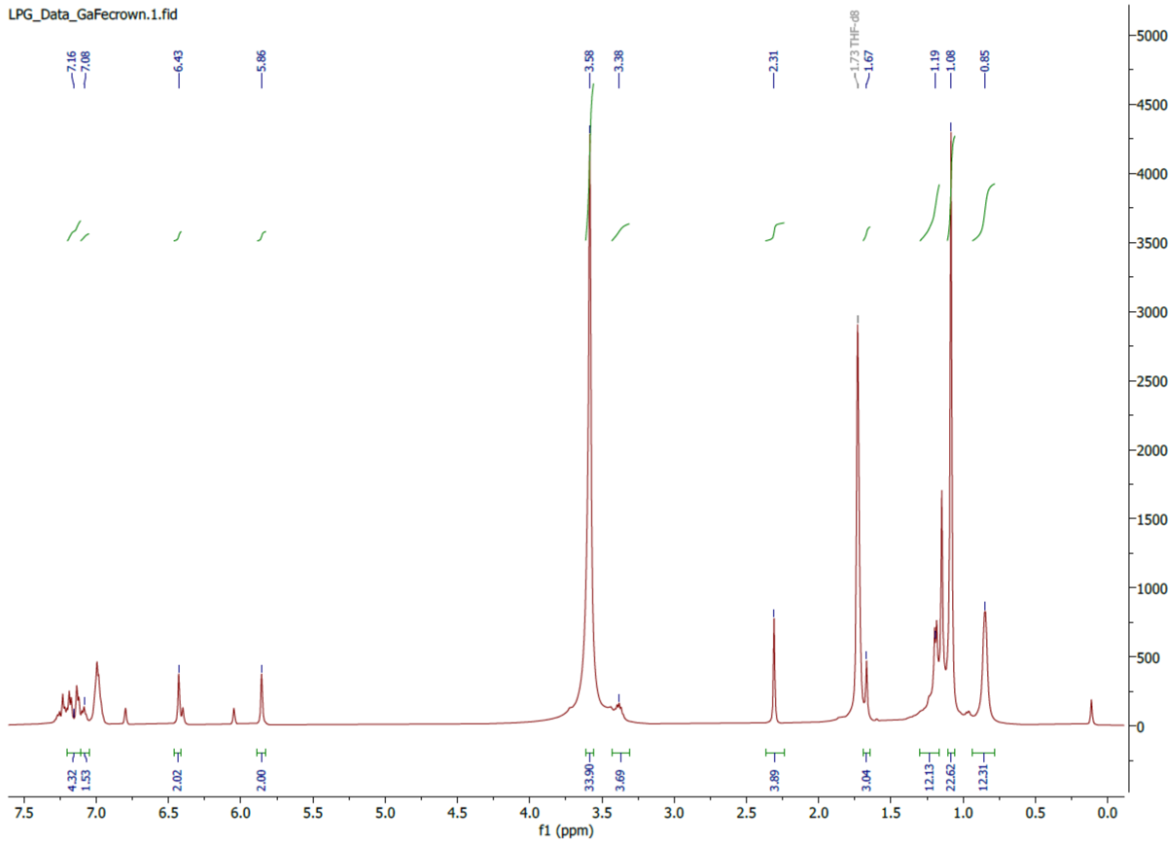

Figure s6: ^{1}H NMR spectrum of **3-crown**

Compound 4

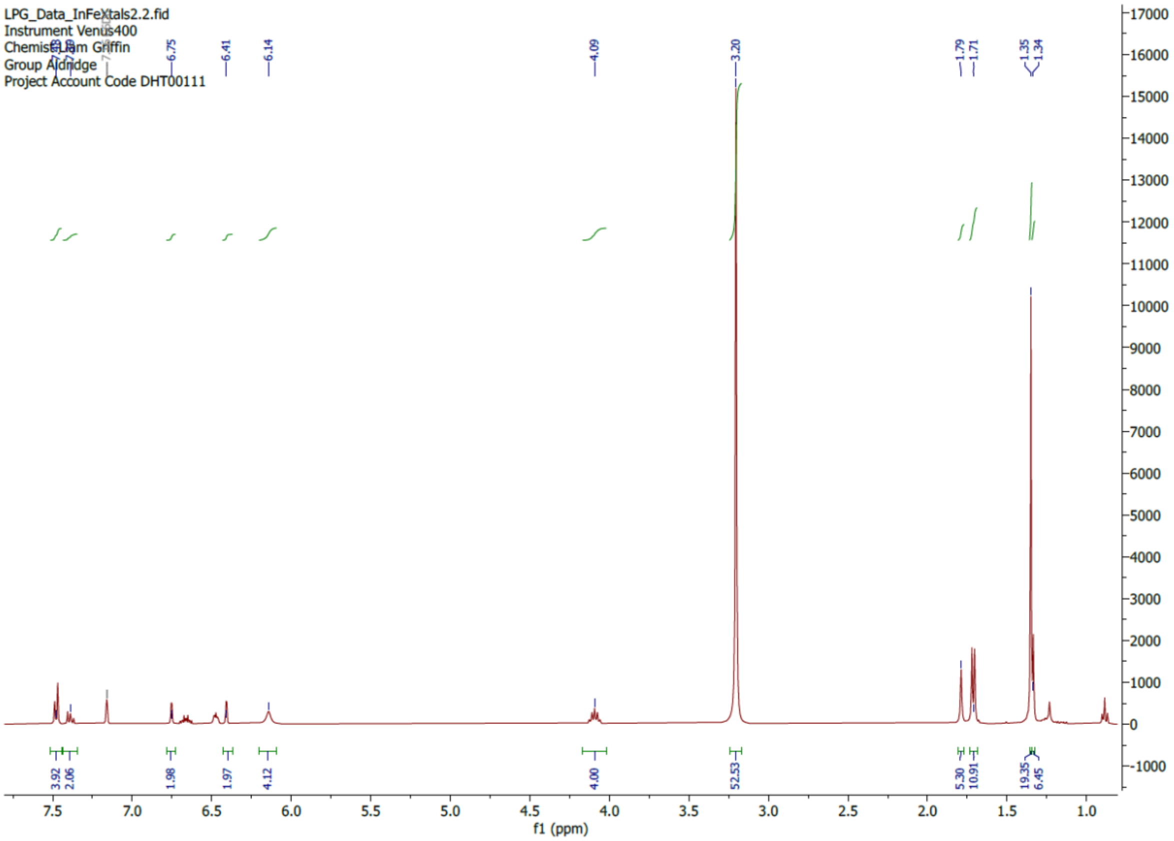

Figure s7: ^{1}H NMR spectrum of **4**

5. **IR spectra of novel compounds**

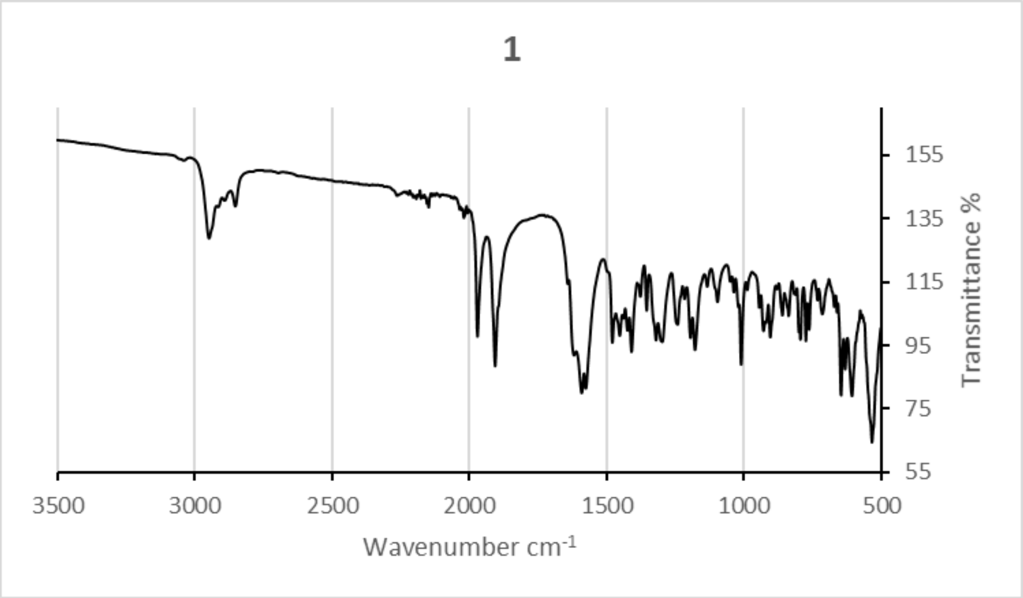

Figure s8: IR spectrum of **1**

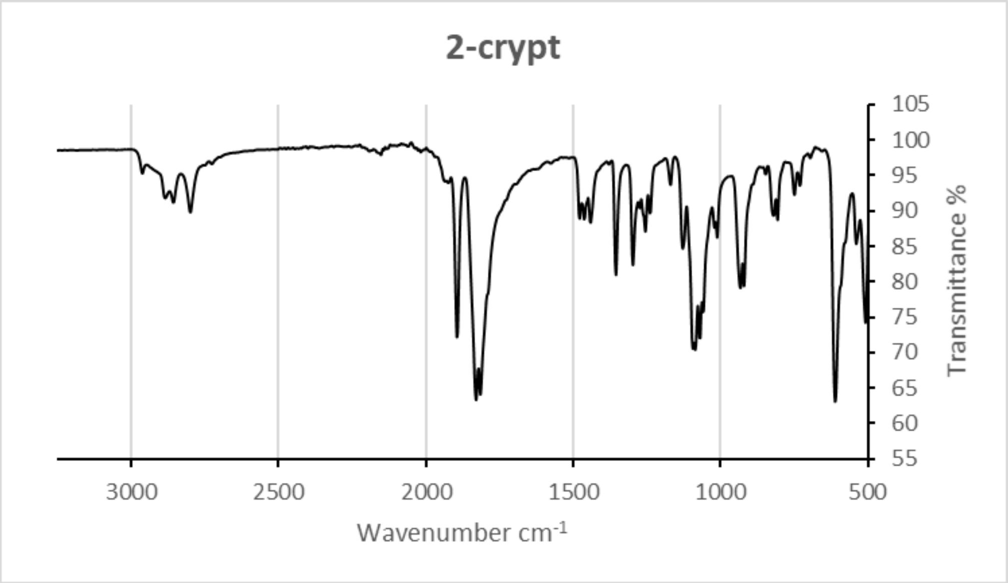

Figure s9: IR spectrum of **2-crypt**

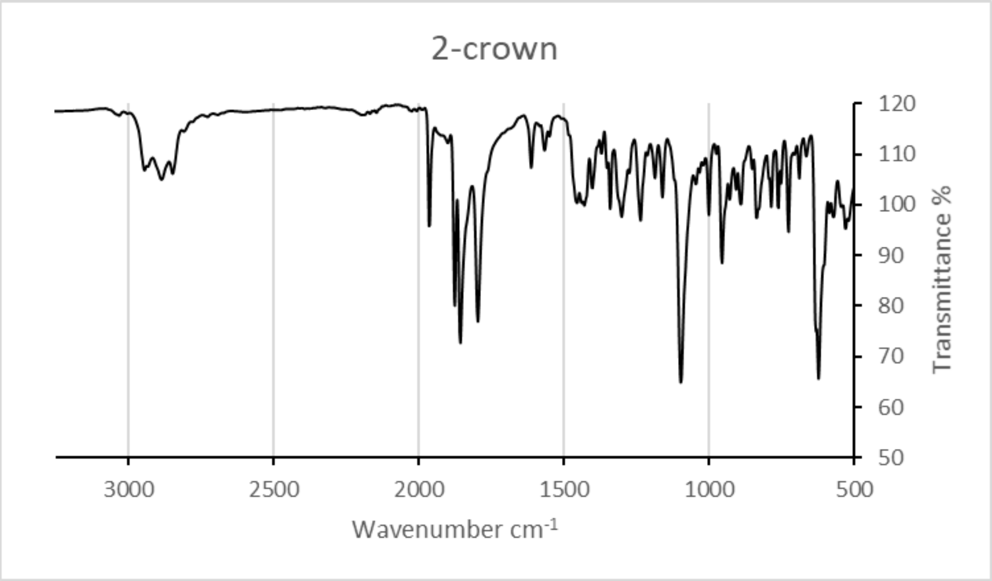

Figure s10: IR spectrum of **2-crown**

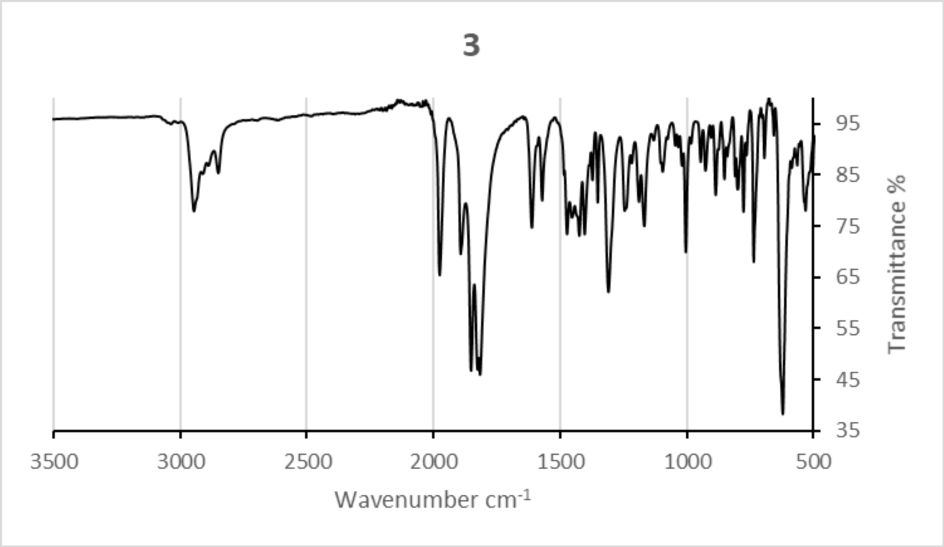

Figure s11: IR spectrum of **3**

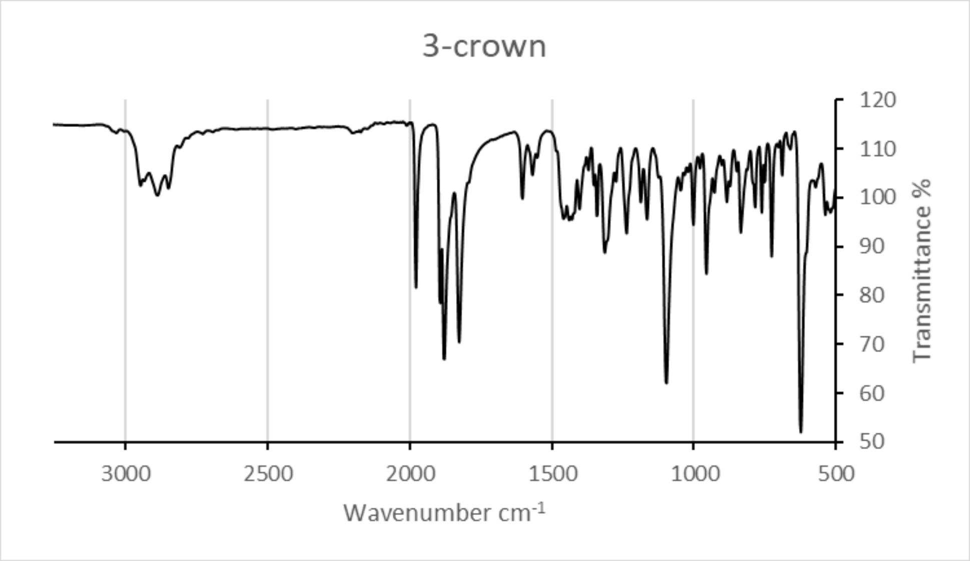

Figure s12: IR spectrum of **3-crown**

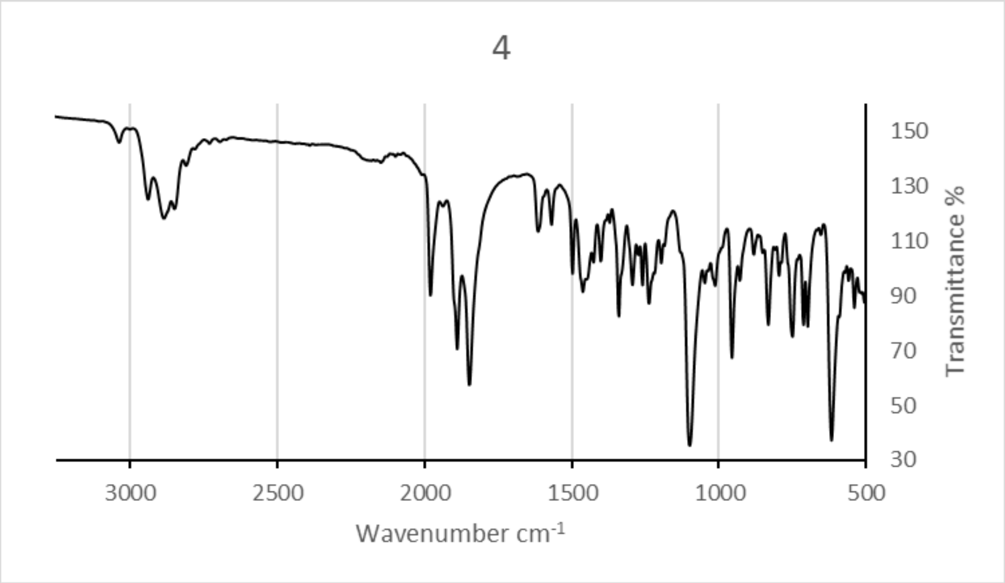

Figure s13: IR spectrum of **4**

6. Mössbauer spectra of novel compounds

All samples were prepared in a Delrin Mössbauer cup in a glovebox under nitrogen-atmosphere and were frozen under nitrogen to 77K within the glovebox. Each sample was then loaded into the Mössbauer sample compartment under liquid nitrogen. ^{57}Fe Mössbauer measurements were performed using a SEE Co. MS4 Mössbauer spectrometer integrated with a Janis SVT-400T cryostat for measurements at 80 K. The Mössbauer spectra were fit using WMoss See Co. Errors of the fit analysis were as follows: $\delta \pm 0.02$ mm/s, $\Delta E_Q \pm 3\%$, and quantitation ±3% (e.g., 50 ± 3%).

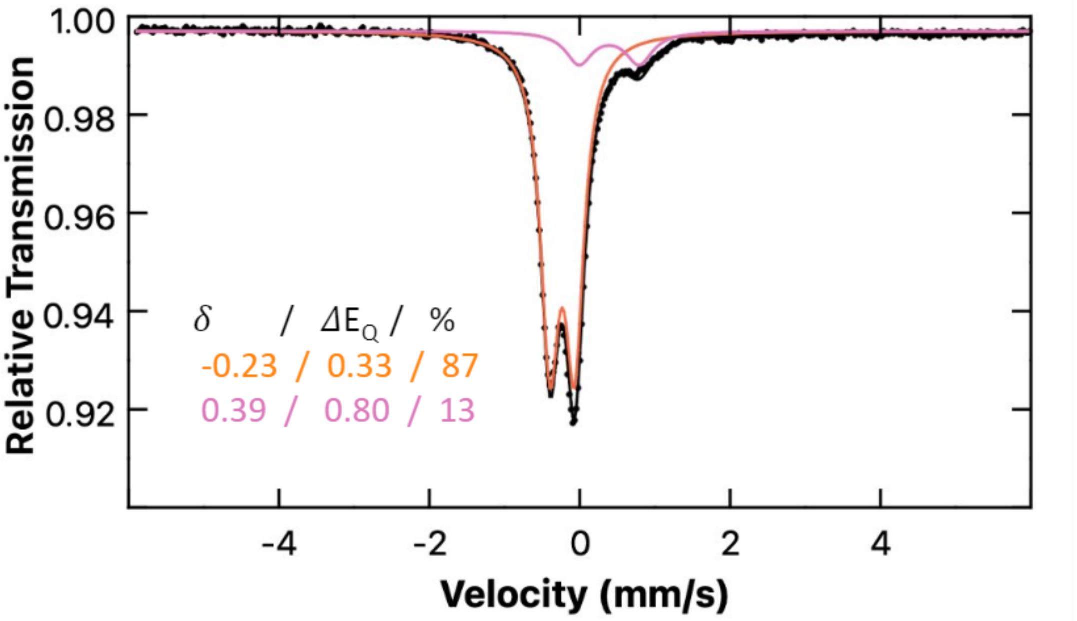

Figure s14: Zero field 80 K ^{57}Fe Mössbauer spectrum of **1** (orange, 87%): δ = -0.23 mm/s, $|\Delta E_Q|$ = 0.33 mm/s 3; (pink, 13%): δ = 0.39 mm/s, $|\Delta E_Q|$ = 0.80 mm/s.

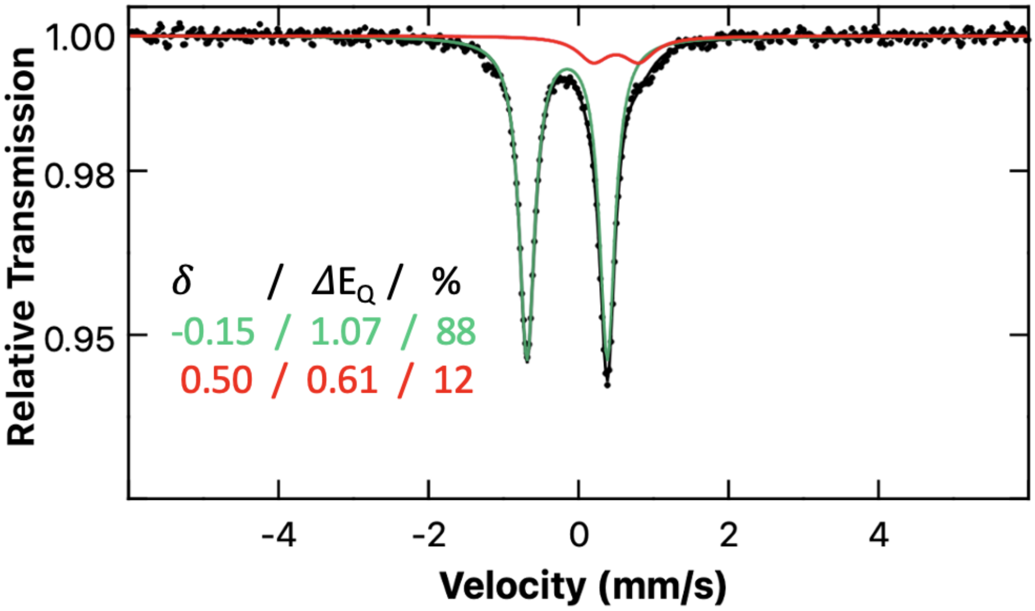

Figure s15: Zero field 80 K ^{57}Fe Mössbauer spectrum of **2-crown** (green, 88%): δ = -0.24 mm/s, $|\Delta E_Q|$ = 2.08 mm/s, (red, 12%): δ = 0.50 mm/s, $|\Delta E_Q|$ = 0.61 mm/s

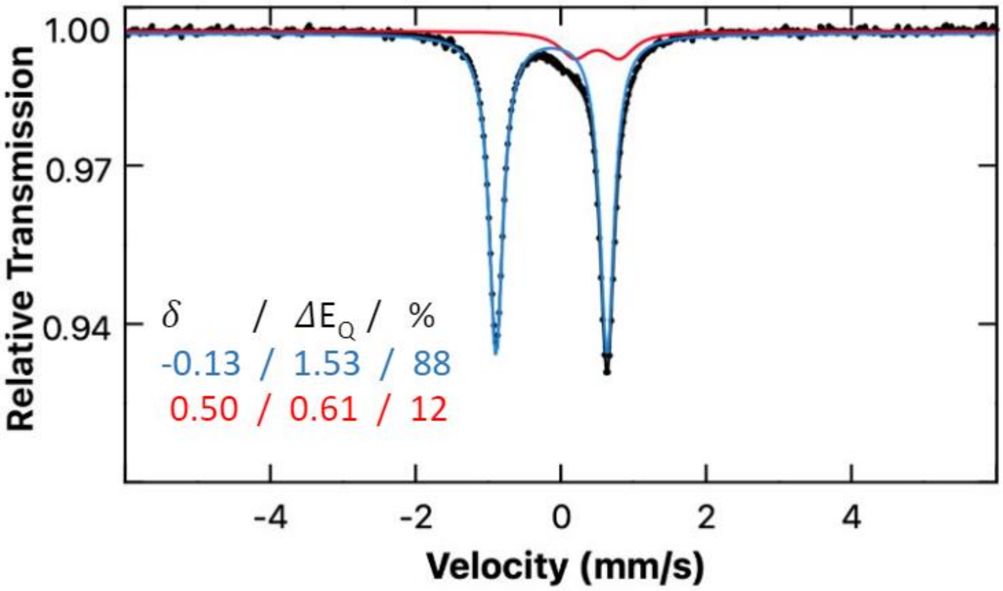

Figure s16: Zero field 80 K ^{57}Fe Mössbauer spectrum of **3-crown** (blue, 88%): δ = -0.13 mm/s, $|\Delta E_Q|$ = 1.53 mm/s, (red, 12%): δ = 0.50 mm/s, $|\Delta E_Q|$ = 0.61 mm/s

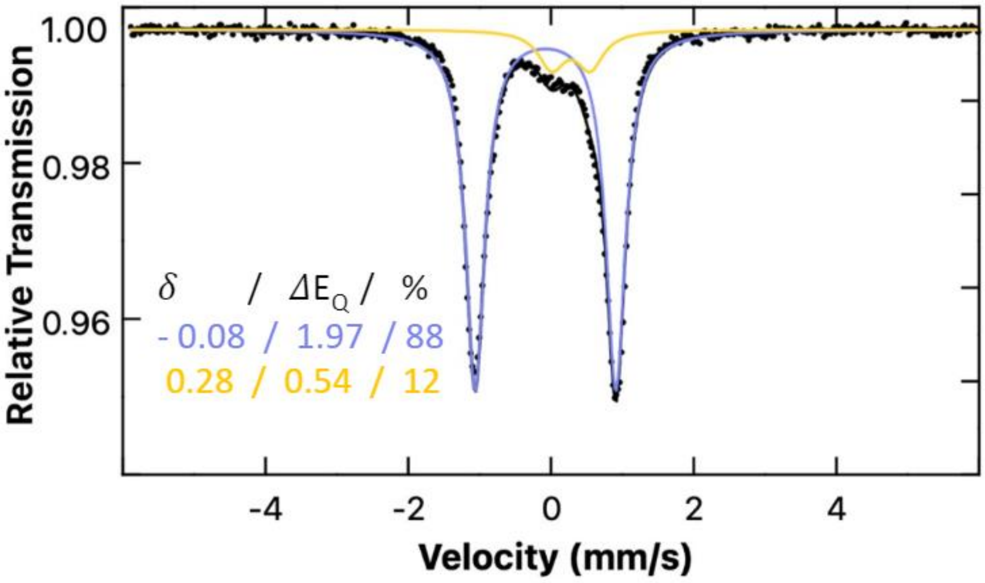

Figure s17: Zero field 80 K ^{57}Fe Mössbauer spectrum of **4** (purple, 88%): δ = -0.08 mm/s, $|\Delta E_Q|$ = 1.97 mm/s, (yellow, 12%): δ = 0.28 mm/s, $|\Delta E_Q|$ = 0.54 mm/s

7. Computational details

Gas phase geometry optimizations and frequency analyses were carried out using the ORCA (5.0.4) software package,[s8, s9] using the R2-Scan-3C method.[s10] The optimized structures were confirmed to be minima on the potential energy surface by the absence of imaginary frequencies. Single point calculations were performed using the ωB97X-D4 functional and Def2-TZVP basis set.[s11-s13] Natural bonding orbital (NBO) analyses were carried out using the NBO 7.0 program.[s14, s15] Atoms in molecules (AIM) and Non-Covalent Interactions (NCI) analyses were conducted using Multiwfn software package.[s16] AIM bonding classifications have been made in accordance with the literature precedent.[s17] All iso-surfaces have been rendered at 0.05, unless otherwise stated.

Compound 1

HOMO

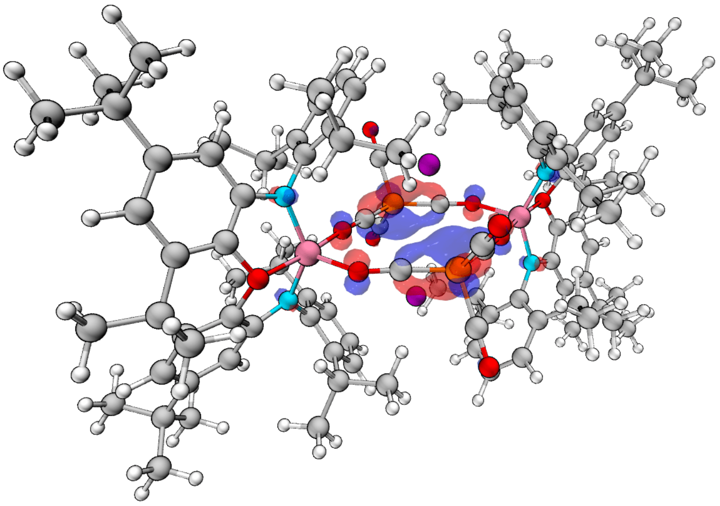

Figure s18: HOMO of **1**

LUMO

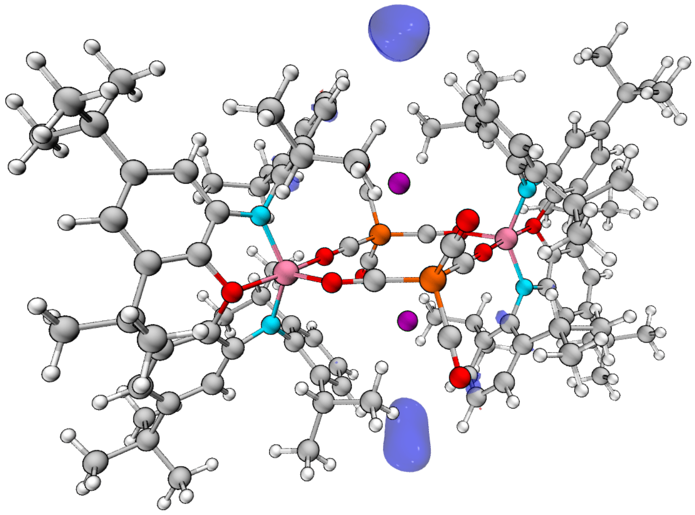

Figure s19: LUMO of **1**. Isosurfaces rendered at 0.02

Compound 2-crypt

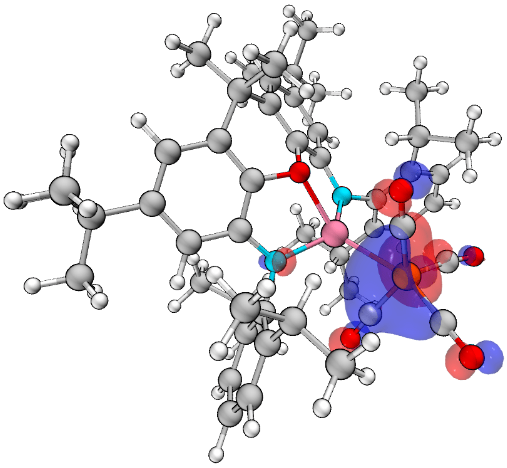

Figure s20: HOMO of **2-crypt**

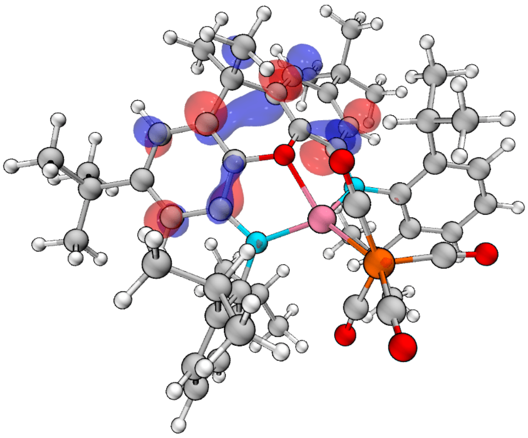

Figure s21: LUMO of **2-crypt**

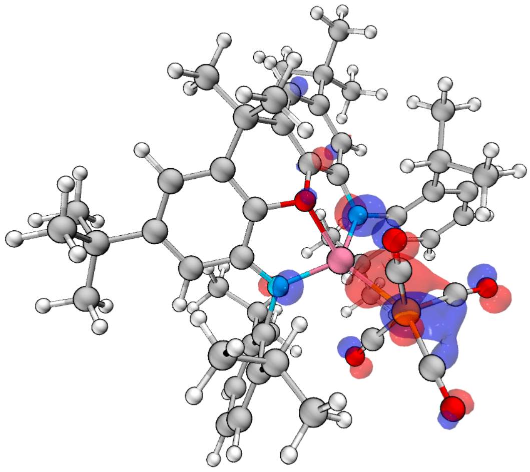

Figure s22: HOMO-1 of **2-crypt**

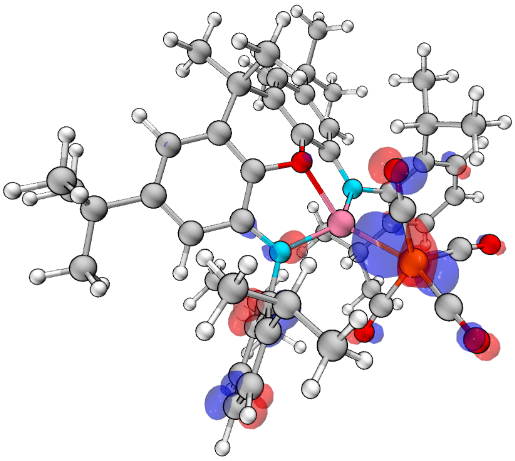

Figure s23: HOMO-6 of **2-crypt**

NCI-plot

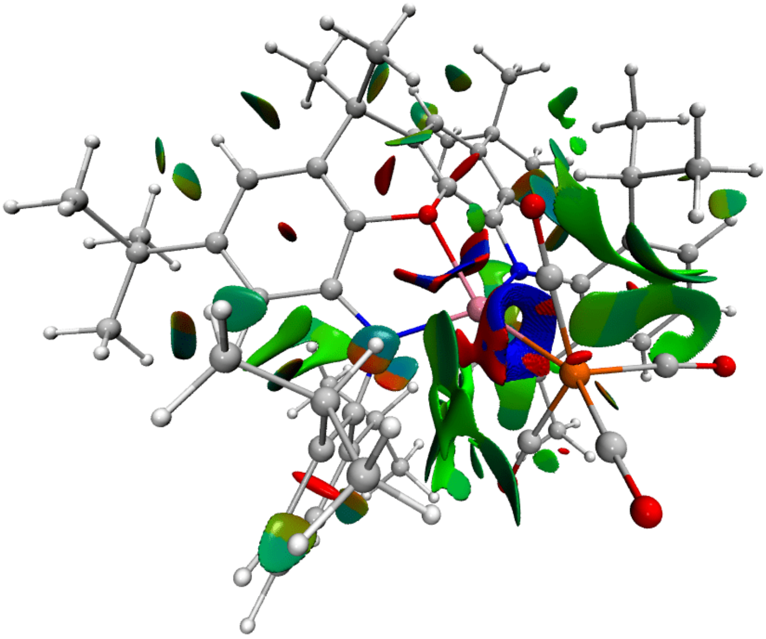

Figure s24: NCI plot of **2-crypt**. Blue regions indicate strongly attractive interactions, red regions indicate strongly repulsive interactions

Compound 2-crown

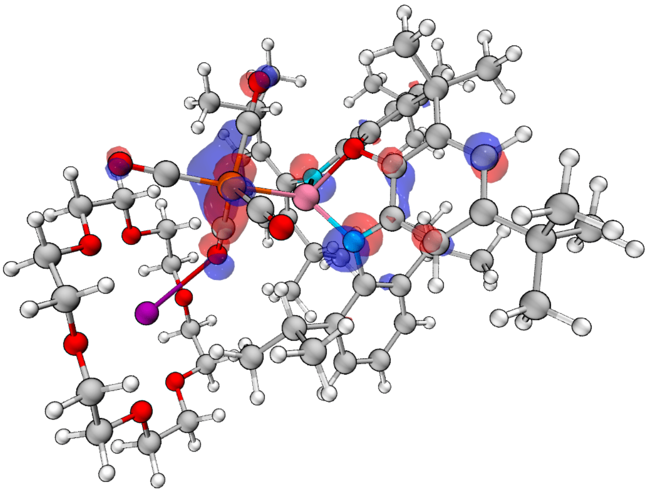

Figure s25: HOMO of **2-crown**

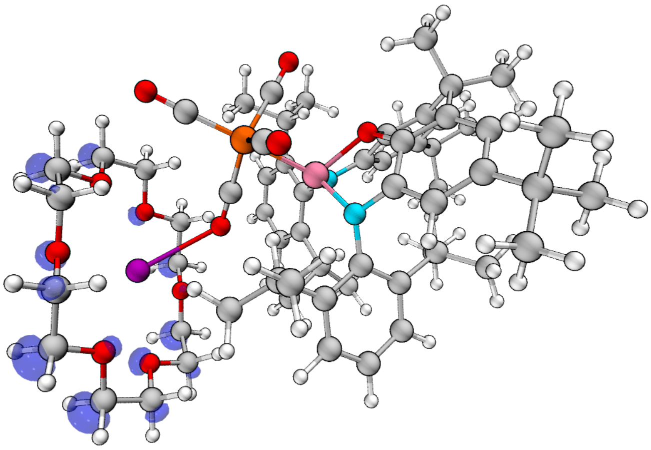

Figure s26: LUMO of **2-crown**. Isosurfaces rendered at 0.02.

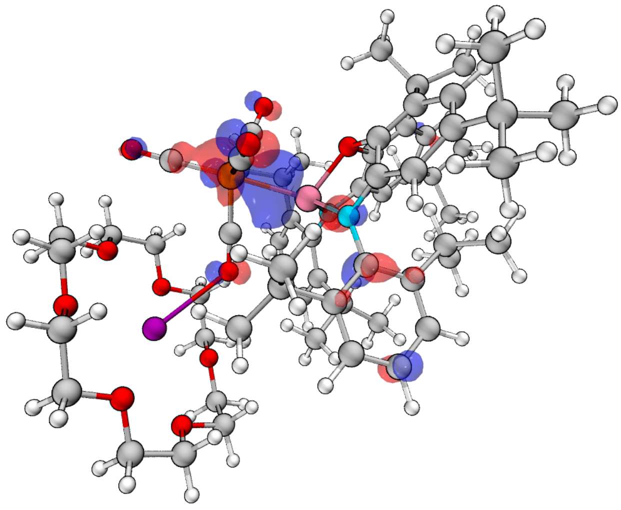

Figure s27: HOMO-4 of **2-crown**

Compound 3-crown

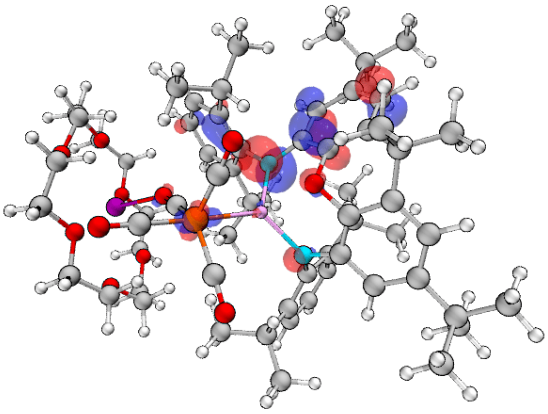

Figure s28: HOMO of **3-crown**

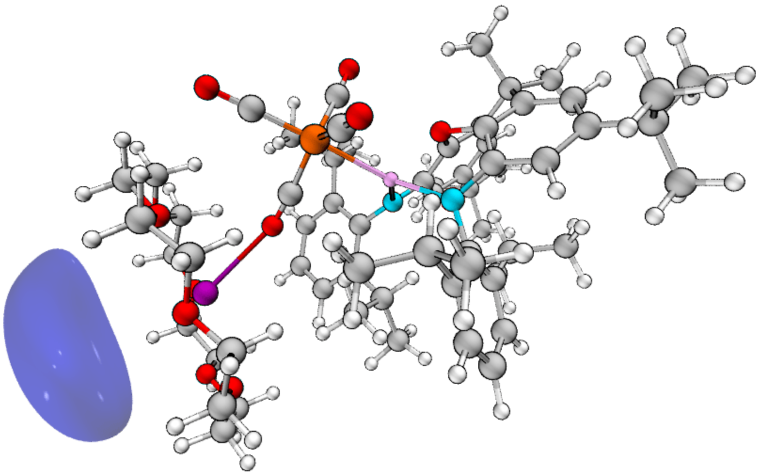

Figure s29: LUMO of **3-crown**. Isosurfaces rendered at 0.025

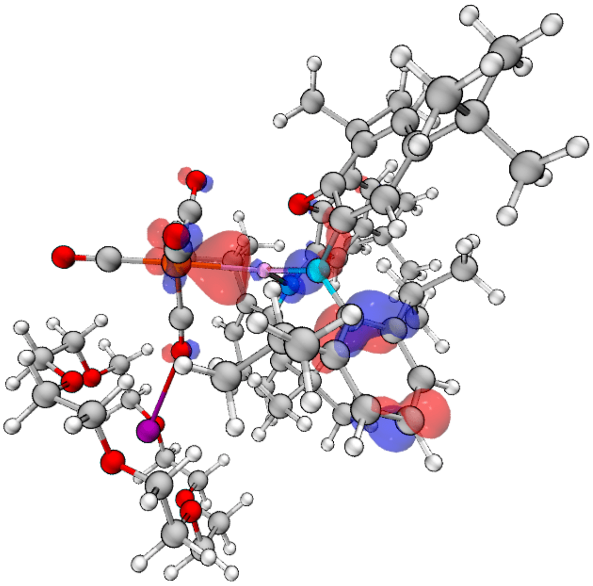

Figure s30: HOMO-4 of **3-crown**

Compound 4

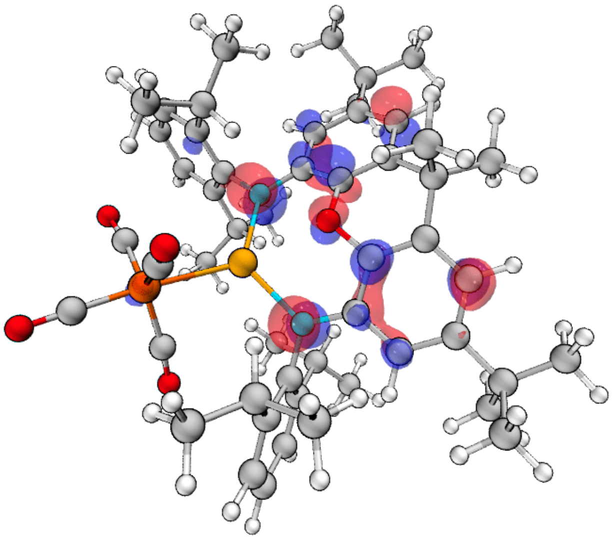

Figure s31: HOMO of **4**

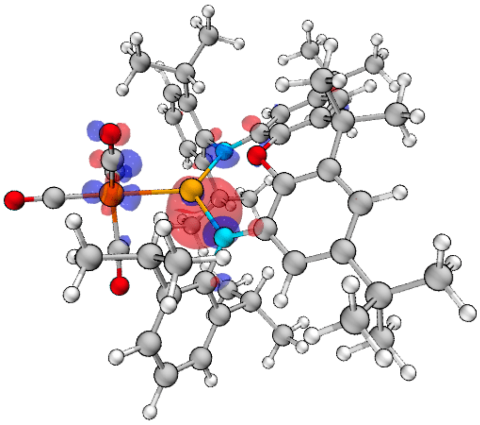

Figure s32: LUMO of **4**

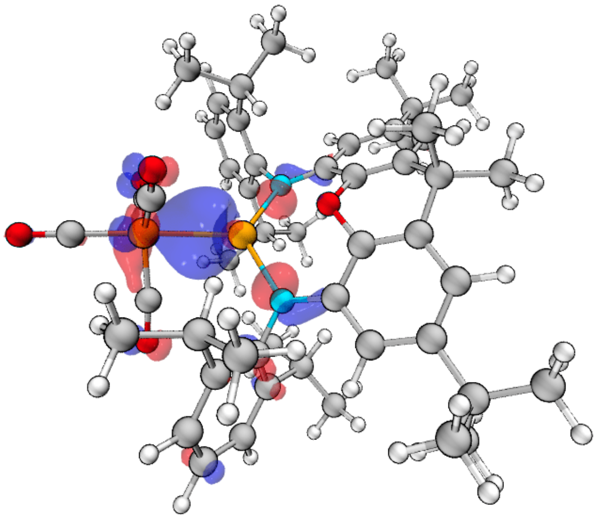

Figure s33: HOMO-4 of **4**

QT-AIM

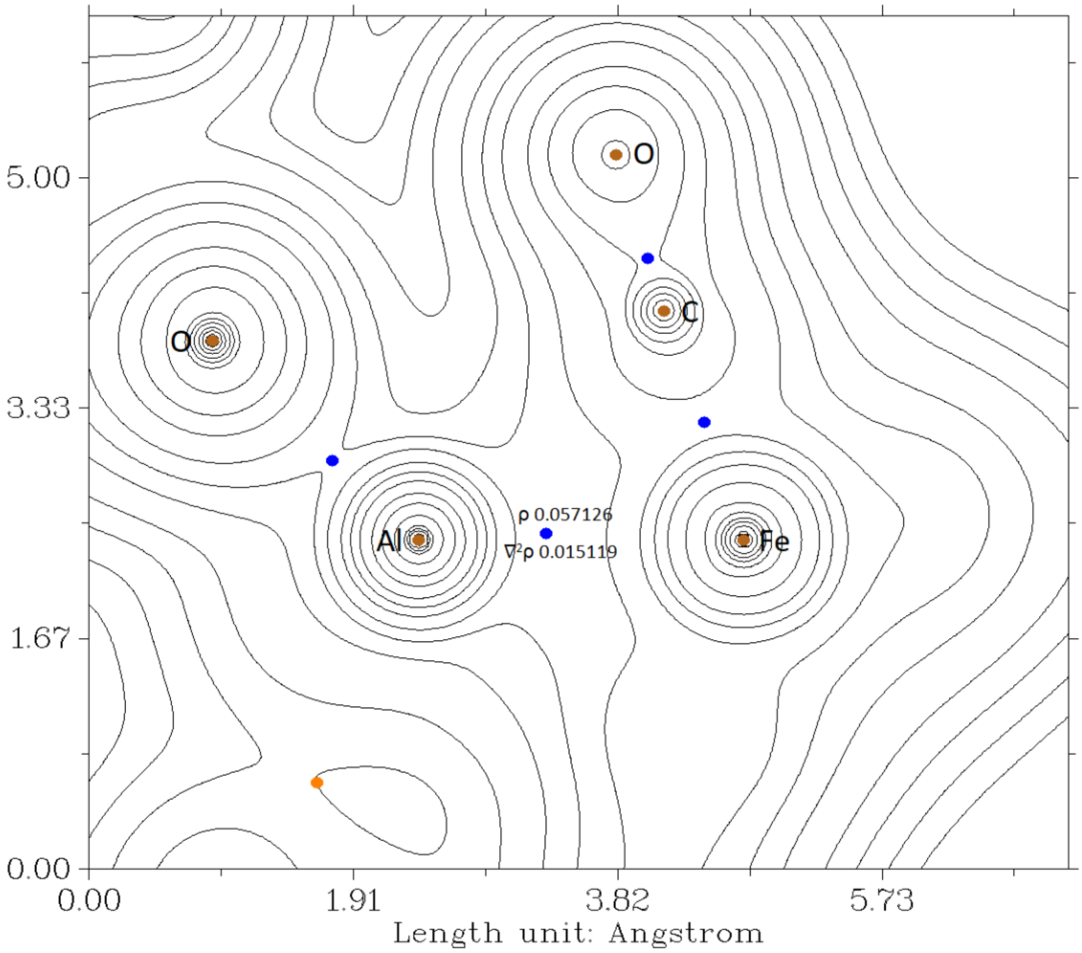

Figure s34: QT-AIM contour map of electron density in the Al–Fe region of **2-crypt**

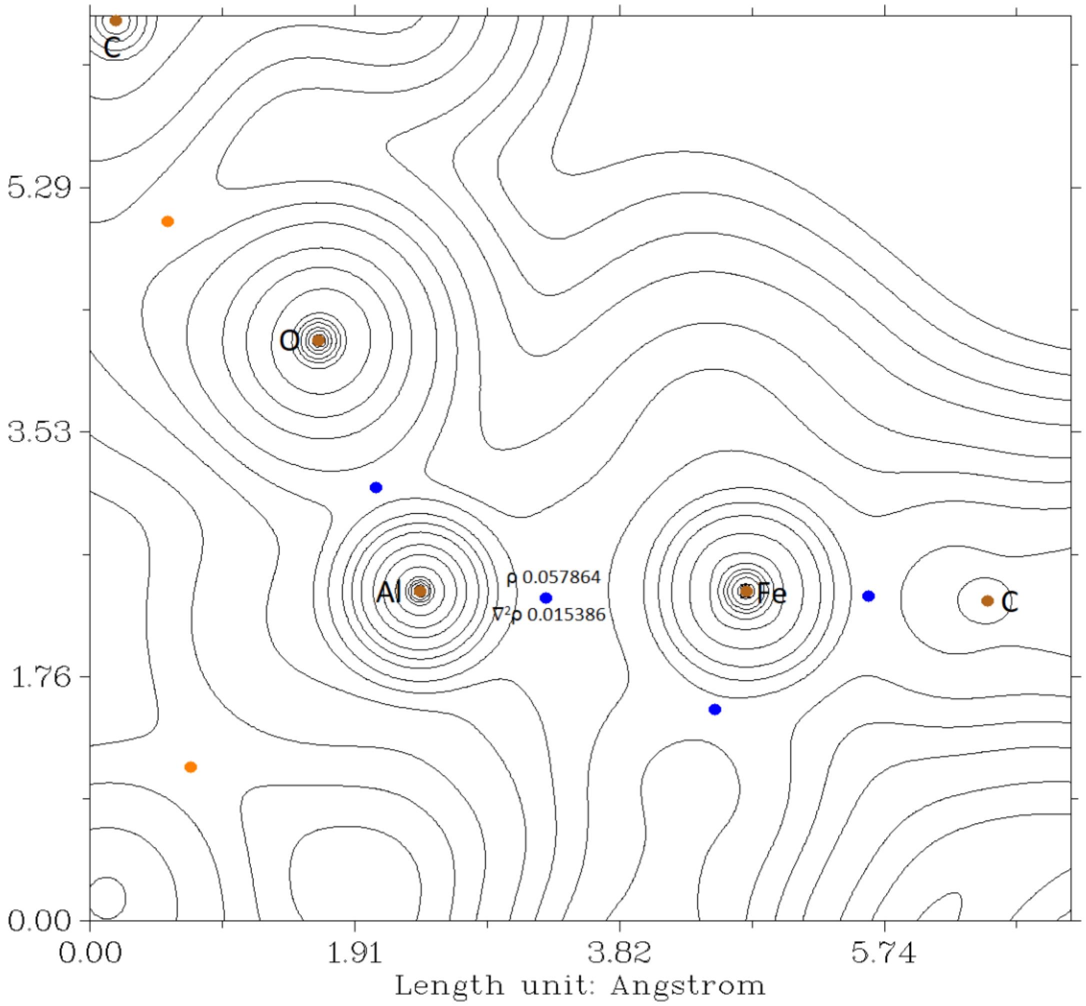

Figure s35: QT-AIM contour map of electron density in the Al–Fe region of **2-crown**

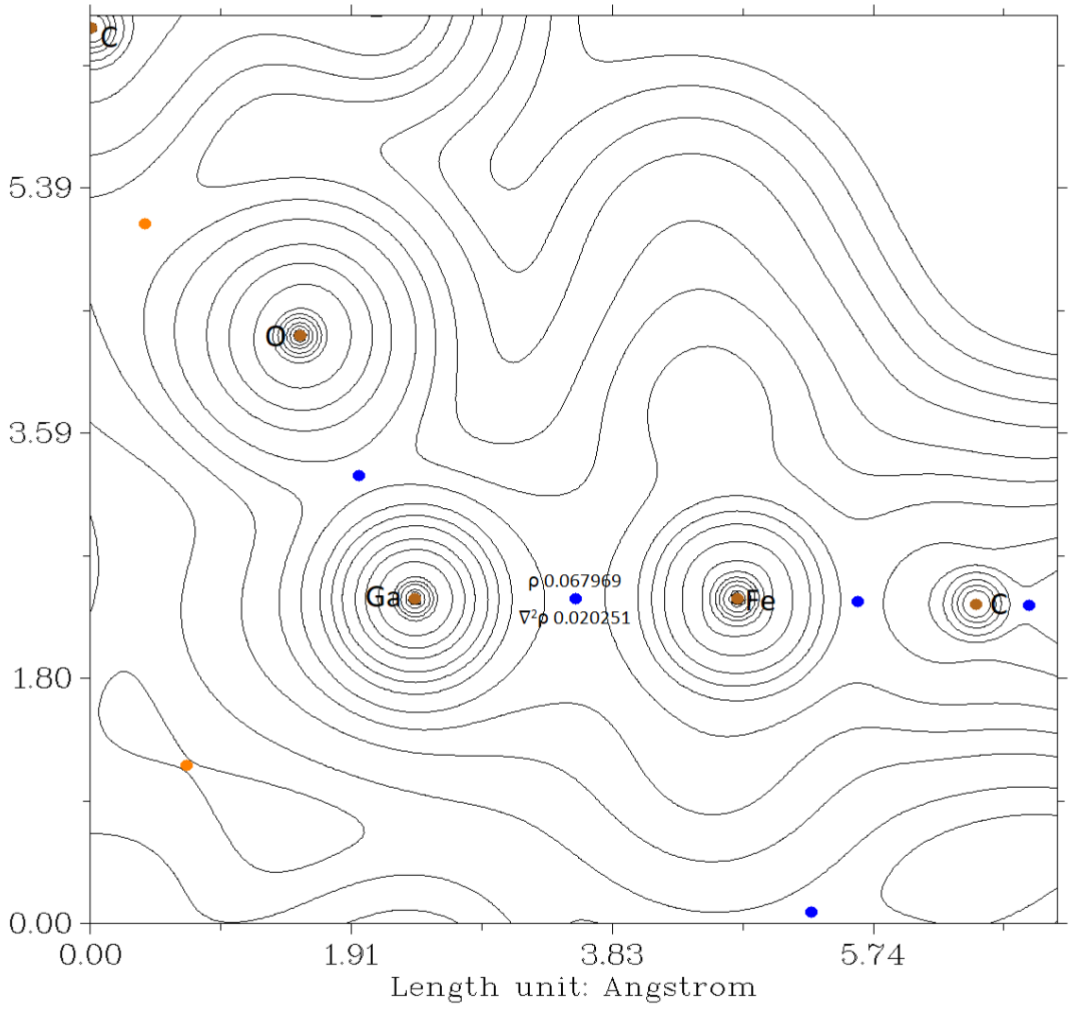

Figure s36: QT-AIM contour map of electron density in the Ga–Fe region of **3-crown**

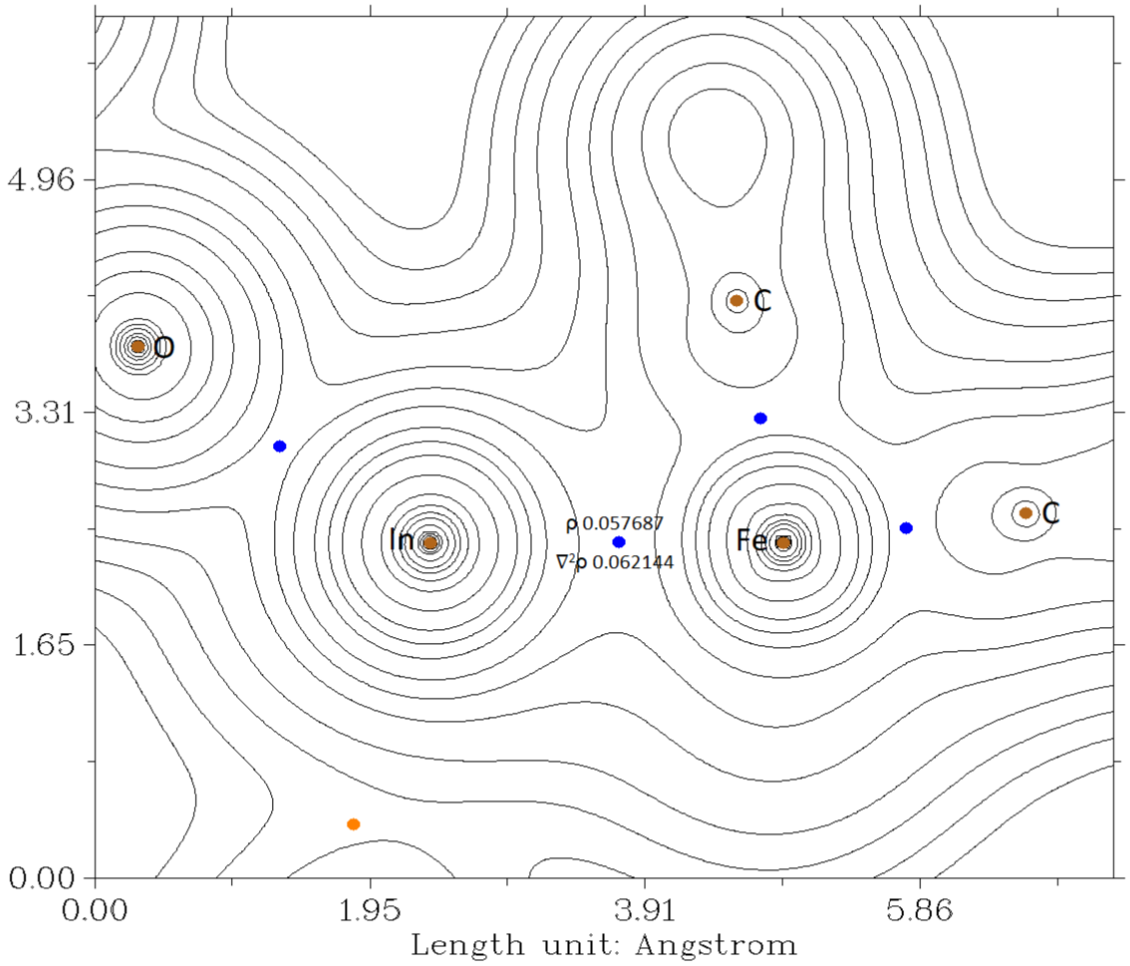

Figure s37: QT-AIM contour map of electron density in the In–Fe region of **4**

QTAIM parameters and WBIs

Table s2: QTAIM parameters associated with E–Fe bonds

	**2-crypt**	**2-crown**	**3-crown**	**4**
**ρ**	**0.057126**	**0.057864**	**0.067969**	**0.057687**
**$\nabla^2\rho$**	**0.015119**	**0.015386**	**0.020251**	**0.062144**
V	-0.052513	-0.053231	-0.057701	-0.050277
G	0.028143	0.028539	0.031382	0.032906
E	-0.024367	-0.024692	-0.023190	-0.017370
ε	0.967191	0.413192	0.071881	0.117847
ELF	0.427464	0.431169	0.517386	0.360771
WBI	Al–Fe 0.3368, Al–C1 0.3342, Al–C2 0.3752	0.3315	0.4428	0.4690

8. XYZ coordinates of calculated structures

1: -9149.842087326791 E_h				2-cryptand: -3974.980414229571 E_h			
Fe	14.523483	4.408251	8.101838	Fe	9.266396	4.078529	3.396226
K	15.119176	4.015754	4.671837	Al	10.377921	4.592930	5.400925
Al	15.425897	8.387874	6.119628	O	12.229864	5.363161	5.924707
O	14.204811	9.914186	6.582684	O	7.260260	6.059679	2.626510
O	14.539229	7.201452	7.229694	O	7.719580	2.611881	5.404047
O	16.374142	2.249200	7.398644	N	11.138447	3.086886	6.336153
N	16.628127	9.400483	7.168407	O	8.936520	2.235031	1.150816
N	14.495828	8.535484	4.483766	N	9.851770	6.292560	6.120591
O	11.757382	3.630957	7.548988	O	11.980458	4.985403	2.740897
O	14.409974	4.540539	11.024246	C	13.034959	4.451100	6.626720
C	14.853039	10.947348	7.277657	C	12.417851	3.224933	6.833778
C	13.616622	9.601205	4.352470	C	12.129705	6.568927	6.637384
C	14.236467	12.165351	7.456383	C	13.167980	2.284690	7.568349
C	12.774569	11.572899	5.571448	H	12.736601	1.304712	7.728898
C	13.454954	10.383641	5.491989	C	10.632591	1.750277	6.417639
C	13.527587	6.663585	3.223067	C	10.831779	7.047168	6.738420
C	16.162012	10.634672	7.611257	C	8.705232	7.034055	5.699632
C	17.828608	8.924092	7.777981	C	10.675295	8.244863	7.458752
C	14.567183	7.602337	3.407857	H	9.678448	8.662943	7.533430
C	12.917489	10.025719	3.217362	C	14.209701	4.873309	7.208971
H	13.010476	9.445985	2.304041	C	13.049838	8.293218	7.952006
C	15.599610	3.153450	7.684133	H	13.894488	8.763359	8.443006
C	14.985861	13.135996	8.123663	C	14.916735	3.922348	7.947312
H	14.551121	14.112728	8.299739	H	15.852667	4.200642	8.418282
C	12.114616	11.982965	4.405063	C	7.440056	6.742040	6.245803
H	11.567668	12.915712	4.414171	C	14.409998	2.621103	8.106561
C	16.296680	12.872990	8.554394	C	11.059486	0.781398	5.486436
C	19.031339	8.848305	7.030782	C	9.773709	1.398093	7.477088
C	16.878005	11.629896	8.292237	C	8.852218	8.063170	4.741746
H	17.905434	11.424892	8.567371	C	8.091071	5.323437	2.973189
C	12.817110	12.326752	6.904376	C	11.771598	8.868443	8.057068
C	17.067131	13.972732	9.294459	C	8.385260	3.183790	4.624376
C	12.174276	11.216323	3.235482	C	13.246130	7.112731	7.232181
C	17.806887	8.547893	9.143708	C	11.613968	10.176672	8.841609
C	14.597754	6.013742	7.555727	C	11.947159	1.124143	4.303947
C	19.126330	9.414850	5.619328	H	11.947569	2.213528	4.196397
H	18.311722	8.975303	5.031176	C	9.391123	0.064115	7.616376
C	15.684011	7.619071	2.541837	H	8.731367	-0.224168	8.429176
C	12.436915	13.797412	6.741686	C	10.651548	-0.541871	5.667188
H	11.414510	13.889612	6.363757	H	10.974990	-1.298289	4.956952
H	13.114107	14.314753	6.054684	C	9.082879	2.953322	2.054460
H	12.465291	14.305193	7.710131	C	10.168508	8.361543	4.038176
C	14.808736	5.653513	1.423544	H	10.916528	7.640910	4.381168
H	14.914206	4.886332	0.661130	C	14.563840	6.353878	7.049585
C	11.498507	11.678987	1.938199	C	7.731869	8.820468	4.402359
C	13.673913	5.694377	2.223534	H	7.831069	9.612395	3.664047
H	12.879207	4.968003	2.062268	C	15.235131	1.598097	8.896627
C	18.976275	8.037498	9.719442	C	6.344445	7.511425	5.855705

H	18.974335	7.757788	10.770728	H	5.361306	7.291065	6.258919
C	16.576344	8.687544	10.029120	C	15.089087	6.602062	5.615088
H	15.746216	9.054289	9.418612	H	14.359737	6.288951	4.863113
C	11.826445	11.653042	7.884121	H	16.012957	6.035506	5.453734
H	11.864057	12.153631	8.857638	H	15.295434	7.668833	5.473102
H	12.070968	10.597039	8.030564	C	9.835237	-0.904909	6.727276
H	10.807097	11.718790	7.489062	H	9.528915	-1.940446	6.851821
C	20.134892	7.883793	8.971843	C	10.057632	8.192280	2.515980
H	21.032524	7.474587	9.428067	H	9.758820	7.175107	2.254939
C	20.156891	8.289582	7.642158	H	11.030241	8.392916	2.051651
H	21.075080	8.178347	7.077715	H	9.326336	8.886560	2.086606
C	15.795514	6.620355	1.574452	C	9.288981	2.436392	8.471680
H	16.663913	6.592482	0.925539	H	9.301436	3.408060	7.963512
C	12.243491	6.694590	4.036683	C	6.487871	8.555546	4.952297
H	12.310164	7.520321	4.751604	H	5.623030	9.143968	4.657762
C	16.349474	14.279797	10.622595	C	10.165048	10.679290	8.858338
H	15.324486	14.624589	10.454306	H	9.791597	10.875034	7.847214
H	16.884533	15.062398	11.173534	H	10.111266	11.616536	9.423686
H	16.302982	13.384725	11.252814	H	9.493059	9.959683	9.338236
C	18.511843	13.567152	9.610826	C	7.291075	5.637211	7.274518
H	18.559841	12.688517	10.263602	H	7.932826	4.806565	6.956395
H	19.013732	14.389893	10.130682	C	15.624202	6.800193	8.053250
H	19.080445	13.353318	8.699011	H	15.854793	7.861243	7.914678
C	16.690894	8.753288	2.595106	H	16.553609	6.243711	7.896349
H	16.707949	9.137363	3.621717	H	15.292500	6.644332	9.085012
C	12.863019	3.914342	7.761690	C	13.395307	0.654847	4.517082
C	18.939394	10.942444	5.614465	H	13.430346	-0.434044	4.651640
H	17.964663	11.250228	5.992245	H	13.848531	1.124294	5.394609
H	19.043515	11.321463	4.591472	H	14.003897	0.907113	3.640597
H	19.712011	11.419317	6.229666	C	11.396695	0.536260	2.998479
C	16.143484	7.341972	10.619885	H	11.910961	0.984052	2.141128
H	15.249542	7.463866	11.239971	H	10.329878	0.741890	2.893535
H	16.921995	6.902378	11.255095	H	11.550403	-0.548836	2.948757
H	15.880886	6.624192	9.837207	C	10.896342	4.623125	3.028909
C	14.467976	4.481159	9.867441	C	12.489257	11.267790	8.195394
C	17.113203	15.246660	8.429282	H	13.549312	10.996521	8.212512
H	17.594007	15.041700	7.466754	H	12.372766	12.217709	8.731955
H	17.684210	16.029712	8.941432	H	12.200705	11.422755	7.149960
H	16.113085	15.641732	8.228845	C	7.786804	6.116743	8.647182
C	16.817260	9.704020	11.155662	H	7.187875	6.971799	8.985007
H	17.603627	9.360138	11.837619	H	8.834182	6.428426	8.603307
H	15.901371	9.837989	11.741335	H	7.697338	5.319877	9.394914
H	17.110100	10.678236	10.756168	C	10.680167	9.771137	4.373790
C	20.440052	9.087633	4.908329	H	9.994475	10.536192	3.989722
H	21.293724	9.551239	5.416798	H	11.661492	9.938709	3.914279
H	20.413145	9.495833	3.894593	H	10.780167	9.910689	5.454107
H	20.620321	8.014931	4.829824	C	7.855333	2.176035	8.942189
C	18.115453	8.349392	2.218748	H	7.792199	1.289449	9.583840
H	18.488933	7.537682	2.849381	H	7.185207	2.036881	8.088418
H	18.776686	9.212796	2.341346	H	7.496805	3.024786	9.534449
H	18.191608	8.035940	1.172151	C	5.871674	5.078165	7.375032

C	12.035920	5.410323	4.842383
H	12.822945	5.273343	5.590018
H	11.997366	4.524192	4.195541
H	11.092094	5.447004	5.395132
C	11.029132	6.954608	3.133935
H	11.159706	7.867669	2.548973
H	10.125671	7.067680	3.742862
H	10.863105	6.122659	2.439721
C	16.215427	9.909162	1.696611
H	16.129264	9.573120	0.656297
H	16.936533	10.733642	1.730901
H	15.244136	10.295235	2.016825
C	12.584515	11.958259	0.880815
H	13.279240	12.728806	1.232518
H	12.125461	12.306138	-0.052153
H	13.165972	11.058995	0.655093
C	10.554061	10.581605	1.414498
H	11.099414	9.673487	1.141125
H	10.029939	10.932911	0.518290
H	9.807093	10.316407	2.170472
C	10.676241	12.959434	2.129445
H	9.878503	12.822030	2.867844
H	10.207002	13.233813	1.178851
H	11.300972	13.802707	2.442691
Fe	18.503398	4.938146	4.847479
K	17.907585	5.330227	8.277643
Al	17.600645	0.958407	6.829014
O	18.821514	-0.568041	6.365971
O	18.487765	2.144796	5.719233
O	16.652541	7.097351	5.549765
N	16.398323	-0.053888	5.780099
N	18.530381	0.810480	8.465052
O	21.269418	5.715489	5.400665
O	18.617177	4.806075	1.925091
C	18.173155	-1.601050	5.670888
C	19.409424	-0.255373	8.596317
C	18.789568	-2.819127	5.492111
C	20.251348	-2.227072	7.377257
C	19.571144	-1.037711	7.456745
C	19.498728	2.682221	9.725925
C	16.864258	-1.288146	5.337211
C	15.197923	0.422654	5.170464
C	18.459079	1.743539	9.541039
C	20.108291	-0.680158	9.731495
H	20.015203	-0.100544	10.644880
C	17.427108	6.192963	5.264812
C	18.040059	-3.789622	4.824743
H	18.474651	-4.766418	4.648663
C	20.911066	-2.637377	8.543689
H	21.457866	-3.570207	8.534549
C	16.729277	-3.526419	4.394019

H	5.867227	4.195534	8.021826
H	5.495532	4.777284	6.392176
H	5.176784	5.807373	7.809122
C	12.060715	9.960403	10.300108
H	11.456097	9.179190	10.773327
H	11.943591	10.887007	10.875792
H	13.109740	9.654707	10.361182
C	10.239319	2.545150	9.673870
H	9.884758	3.311679	10.373926
H	11.253093	2.810469	9.359972
H	10.288026	1.587472	10.207783
C	15.473355	2.117431	10.326988
H	16.055412	1.388983	10.905287
H	14.518261	2.281972	10.837795
H	16.021250	3.064729	10.329415
C	14.540876	0.234088	8.996286
H	15.177249	-0.458167	9.559188
H	14.362213	-0.202206	8.007429
H	13.579819	0.304995	9.517176
C	16.589384	1.391877	8.192084
H	17.161552	2.323349	8.139360
H	16.435232	1.035498	7.167547
H	17.192697	0.650053	8.730438

C	13.995172	0.498626	5.917621	
C	16.148130	-2.283243	4.656187	
H	15.120704	-2.078128	4.381131	
C	20.208838	-2.980793	6.044253	
C	15.958693	-4.626022	3.653884	
C	20.851328	-1.870863	9.713356	
C	15.219731	0.798759	3.804713	
C	18.429235	3.332599	5.393489	
C	13.900064	-0.067842	7.329092	
H	14.714370	0.372078	7.917390	
C	17.342263	1.726783	10.407081	
C	20.588784	-4.451527	6.206850	
H	21.611139	-4.543932	6.584868	
H	19.911441	-4.968824	6.893736	
H	20.560428	-4.959216	5.238356	
C	18.217758	3.692018	11.525747	
H	18.112401	4.459023	12.288354	
C	21.526806	-2.333769	11.010710	
C	19.352542	3.651202	10.725706	
H	20.147354	4.377430	10.887113	
C	14.050394	1.309156	3.228867	
H	14.052337	1.588737	2.177549	
C	16.450337	0.659048	2.919398	
H	17.280240	0.291677	3.529827	
C	21.199714	-2.307149	5.064678	
H	21.162142	-2.807649	4.091113	
H	20.955374	-1.251095	4.918294	
H	22.219006	-2.373086	5.459850	
C	12.891774	1.463042	3.976419	
H	11.994180	1.872244	3.520116	
C	12.869694	1.057394	5.306147	
H	11.951502	1.168793	5.870553	
C	17.230866	2.725334	11.374644	
H	16.362526	2.753112	12.023641	
C	20.782870	2.651165	8.912357	
H	20.716372	1.825150	8.197743	
C	16.676413	-4.933205	2.325809	
H	17.701335	-5.278152	2.494188	
H	16.141284	-5.715729	1.774830	
H	16.723094	-4.038142	1.695591	
C	14.514082	-4.220177	3.337389	
H	14.466305	-3.341608	2.684510	
H	14.012054	-5.042872	2.817595	
H	13.945473	-4.006117	4.249146	
C	16.335022	0.592917	10.353409	
H	16.317656	0.209338	9.326616	
C	20.163798	5.432148	5.187803	
C	14.087518	-1.595369	7.334151	
H	15.062399	-1.902933	6.956576	
H	13.983317	-1.974284	8.357173	
H	13.315146	-2.072550	6.718880	

C 16.883743 2.004774 2.329388<br>H 17.777554 1.882869 1.709114<br>H 16.105320 2.445086 1.694568<br>H 17.146728 2.721968 3.112475<br>C 18.559031 4.865287 3.081897<br>C 15.912297 -5.899950 4.519044<br>H 15.431471 -5.694898 5.481542<br>H 15.341155 -6.682875 4.006851<br>H 16.912314 -6.295242 4.719543<br>C 16.209269 -0.356814 1.792348<br>H 15.423230 -0.012354 1.110305<br>H 17.125246 -0.490893 1.206840<br>H 15.915939 -1.331098 2.191324<br>C 12.586035 0.258938 8.039747<br>H 11.732675 -0.205040 7.531094<br>H 12.612853 -0.149190 9.053514<br>H 12.405318 1.331568 8.118152<br>C 14.910671 0.997098 10.730273<br>H 14.537395 1.809390 10.100261<br>H 14.249100 0.134022 10.607211<br>H 14.834832 1.309851 11.777105<br>C 20.990249 3.935141 8.106139<br>H 20.203493 4.071495 7.358102<br>H 21.028277 4.821598 8.752564<br>H 21.934298 3.898563 7.553756<br>C 21.997257 2.391720 9.815230<br>H 21.866839 1.478840 10.400503<br>H 22.900762 2.278619 9.206373<br>H 22.163104 3.223937 10.509172<br>C 16.810292 -0.563546 11.251251<br>H 16.896726 -0.228032 12.291714<br>H 16.088951 -1.387810 11.216699<br>H 17.781421 -0.949745 10.930698<br>C 20.440556 -2.612991 12.067858<br>H 19.745787 -3.383400 11.715939<br>H 20.899373 -2.961028 13.000883<br>H 19.859192 -1.713656 12.293538<br>C 22.471308 -1.236579 11.534702<br>H 21.926046 -0.328397 11.808053<br>H 22.995192 -1.588046 12.430986<br>H 23.218474 -0.971428 10.778909<br>C 22.348934 -3.614311 10.819496<br>H 23.146792 -3.476961 10.081219<br>H 22.817992 -3.888825 11.770141<br>H 21.724143 -4.457484 10.506102	
**2-crown: -5498.661744725587 E_h**	**3-crown : -7180.985061094257 E_h**
Fe 5.080323 4.122431 14.419651<br>K 5.848076 8.806730 15.942223<br>Al 2.824683 4.252903 13.753761<br>O 2.172307 2.557518 13.044721<br>O 6.824930 8.863691 13.249790	Ga 9.171422 18.260465 20.754403<br>Fe 9.125540 16.392534 19.311073<br>K 14.421586 15.785270 19.100205<br>O 7.533088 19.561274 20.507784<br>O 13.928336 15.466917 16.277506

O	5.185519	2.333395	12.104419	N	8.519296	18.319238	22.619266
O	4.223424	2.551394	16.736264	O	12.561788	13.909436	18.179221
O	7.999820	7.260195	15.243795	O	7.455617	18.144161	17.663850
O	4.501538	10.318728	14.039666	N	10.148140	19.889698	20.208321
N	2.272559	4.979744	12.087049	O	15.883932	17.281159	17.262773
O	4.177670	11.020904	16.759665	O	15.424702	15.517687	21.703140
N	1.445609	4.007265	15.057056	O	17.058389	16.777167	19.765275
O	7.619219	7.809743	17.972130	O	7.548345	14.940769	21.312806
O	5.358584	9.335725	18.717394	C	9.790776	22.339054	19.889111
O	4.447784	6.969097	14.518211	H	10.857241	22.510280	19.772891
O	7.969146	4.159360	14.887002	C	6.427687	18.798771	23.879728
C	5.132849	3.067387	13.007973	H	6.778937	18.231019	24.736874
C	1.560817	4.054825	11.328439	O	12.003965	16.820698	19.145200
C	2.655074	6.184593	11.420088	C	8.892769	23.414702	19.896142
C	-0.665229	3.565202	8.255355	C	6.714368	19.537045	21.649023
C	-1.376952	4.212537	14.081827	C	7.045352	21.882570	20.296470
H	-0.652329	3.444598	13.810889	C	7.234839	18.828309	22.734165
C	1.017319	2.685289	15.085031	C	7.974572	20.869222	20.245970
C	0.861405	4.269320	10.134208	C	4.906374	20.406410	22.898080
H	0.877626	5.265222	9.701340	H	4.057798	21.071387	22.988062
C	0.116763	3.241713	9.535764	O	9.204193	14.136020	17.460389
C	1.349621	1.898932	13.984350	C	5.676877	20.435367	21.728367
C	0.064309	1.971996	10.127717	O	13.461748	13.651354	20.812880
H	-0.530826	1.187098	9.680871	C	9.355671	21.019833	20.088162
C	-0.752643	5.135799	15.120836	C	7.526398	23.183379	20.107424
C	3.966476	7.309977	9.720897	H	6.833461	24.012985	20.145156
H	4.718178	7.272715	8.936328	C	5.591973	21.471619	20.597454
C	0.766999	1.707328	11.311116	C	5.245223	19.547264	23.951787
C	3.646010	6.139312	10.408775	C	12.580532	19.696533	20.434115
C	-1.280951	0.127474	16.812554	C	4.378127	19.431410	25.212161
C	1.493624	2.751803	11.823709	C	12.462553	19.447091	21.923506
C	0.573216	4.990099	15.607737	H	11.457645	19.751952	22.226605
C	-1.400715	2.343478	7.691352	C	11.438739	19.898944	19.624061
H	-0.705625	1.533751	7.443780	C	4.739641	22.672291	21.002440
H	-1.924590	2.626411	6.772047	H	5.133951	23.161882	21.898809
H	-2.148587	1.958372	8.393064	H	3.709982	22.358839	21.200165
C	2.030410	7.411585	11.732540	H	4.704861	23.403299	20.188930
C	3.346103	8.515793	10.015401	C	9.449607	24.835510	19.725874
H	3.600196	9.414438	9.458278	C	10.408546	20.103562	17.269734
C	4.385653	4.865843	10.043952	H	9.491300	19.988501	17.852404
H	4.006758	4.055744	10.669325	C	5.181807	19.867324	26.451852
C	0.798459	0.385406	12.102116	H	6.088064	19.267933	26.581363
C	-1.068932	7.033503	16.607919	H	4.571409	19.753537	27.355673
H	-1.705178	7.826548	16.991533	H	5.481044	20.917980	26.370842
C	-1.750167	4.929876	12.780282	C	8.192438	15.545604	20.559975
H	-2.441423	5.762089	12.954791	C	9.342350	18.379841	23.782281
H	-2.233127	4.222033	12.096721	C	9.809251	19.632120	24.266804
H	-0.862658	5.316656	12.275524	C	11.590235	20.048763	18.222662
C	0.174095	2.079330	16.031083	C	4.981292	20.821780	19.333131
H	-0.082241	2.642905	16.920948	H	4.953935	21.551280	18.516433
C	-0.403676	0.834042	15.772547	H	3.959232	20.489543	19.544848

C	2.397797	8.559742	11.024764	H	5.563895	19.958441	19.002806
H	1.906091	9.501734	11.256444	C	8.115539	17.467778	18.339612
C	-1.711166	4.654382	8.562308	C	10.704212	19.637529	25.337137
H	-2.407343	4.314111	9.336575	H	11.071472	20.588265	25.715075
H	-2.286842	4.893868	7.660132	C	8.346675	25.901173	19.706347
H	-1.240227	5.576204	8.917518	H	7.785960	25.923764	20.646992
C	0.665467	0.774140	13.582955	H	7.641219	25.738829	18.884061
C	-1.543101	6.165156	15.635082	H	8.799049	26.889226	19.568359
H	-2.556147	6.288202	15.259711	C	11.126459	18.459259	25.940867
C	4.549480	3.175492	15.811917	H	11.830414	18.492053	26.768294
C	1.055334	5.865461	16.606866	C	13.843631	19.695483	19.840132
C	0.301314	4.078919	7.171699	H	14.721112	19.561723	20.469866
H	0.807777	4.997881	7.481691	C	8.301619	21.567965	24.740982
H	-0.247350	4.294844	6.247279	H	8.814357	21.844453	25.670493
H	1.068014	3.328425	6.950276	H	7.508351	20.858580	24.981961
C	7.441967	7.608637	12.958006	H	7.840344	22.469611	24.321372
H	7.854880	7.613859	11.934962	C	10.397532	25.155264	20.897603
H	6.704763	6.794290	13.028953	H	10.815296	26.163068	20.784454
C	-2.662522	-0.205920	16.218045	H	11.229920	24.446522	20.950071
H	-3.174481	0.702703	15.884666	H	9.860046	25.111432	21.851062
H	-3.286852	-0.695488	16.974706	C	9.104042	15.860094	24.043018
H	-2.588149	-0.883766	15.362692	H	8.295537	16.074839	23.340248
C	-0.202023	0.216226	14.524516	C	10.459038	18.936019	16.271828
H	-0.740672	-0.691728	14.280171	H	11.311804	19.031591	15.587774
C	3.120012	6.958889	17.626030	H	9.544605	18.919655	15.669204
H	2.688220	7.394365	18.535646	H	10.533266	17.978020	16.796030
H	3.065047	7.705954	16.825322	C	10.615496	17.246962	25.503415
H	4.176180	6.758348	17.839525	H	10.908807	16.328022	26.005548
C	5.724778	9.118107	12.374959	C	9.708373	17.189301	24.442862
H	4.977868	8.313677	12.443836	C	12.438177	13.669246	16.783304
H	6.070387	9.173324	11.329093	H	11.443098	13.262930	16.548808
C	-2.608961	3.501098	14.662567	H	13.206791	12.954502	16.442228
H	-2.380960	3.045373	15.629601	C	12.594728	17.955733	22.258938
H	-2.942681	2.709522	13.982008	H	11.825598	17.360905	21.754326
H	-3.441835	4.200497	14.801502	H	12.485417	17.800349	23.338595
C	8.559884	7.369695	13.937919	H	13.573694	17.570836	21.942663
H	9.070760	6.432601	13.666105	C	10.384949	22.001074	23.431131
H	9.289849	8.196674	13.905785	H	10.992963	22.232162	24.313147
C	4.689217	5.807763	14.448282	H	9.907053	22.929805	23.103166
C	2.160877	-0.307093	11.861527	H	11.050470	21.682502	22.626560
H	2.996822	0.332386	12.155742	C	9.298216	20.964602	23.735394
H	2.272063	-0.549042	10.799149	H	8.759060	20.783159	22.802606
H	2.212070	-1.233573	12.443410	C	16.788964	15.832245	21.944884
C	2.428461	5.658771	17.211064	H	17.437074	14.989930	21.649367
H	3.063591	5.184087	16.459180	H	16.955100	16.037436	23.015971
C	0.943105	7.536135	12.780595	C	17.424480	17.898903	18.965704
H	0.754477	6.541252	13.196360	H	18.476695	18.174289	19.150054
C	-0.324507	-0.554157	11.669097	H	16.790211	18.764910	19.216203
H	-0.278480	-1.488809	12.236472	C	9.162761	15.032215	18.199837
H	-0.215048	-0.813000	10.611543	C	12.875877	20.074238	17.683042
H	-1.310229	-0.102241	11.819877	H	12.995672	20.214227	16.610849

C	6.818199	4.160012	14.709834	C	17.148169	17.066454	21.158333
C	0.222276	6.873977	17.090931	H	16.461847	17.889580	21.421019
H	0.586901	7.542864	17.866251	H	18.174085	17.374135	21.421450
C	3.166738	11.246337	15.775248	C	12.593299	14.991377	16.077127
H	2.560645	12.126867	16.046947	H	12.396283	14.859911	15.000385
H	2.500398	10.371328	15.707817	H	11.866229	15.712123	16.483522
C	4.149921	4.466381	8.581297	C	10.325234	21.438996	16.519996
H	4.553439	5.214608	7.888909	H	10.234216	22.275244	17.216781
H	3.082237	4.347641	8.375724	H	9.449674	21.447562	15.861247
H	4.645296	3.512206	8.370354	H	11.214717	21.601689	15.899264
C	6.393239	8.990395	19.633738	C	3.942910	17.962639	25.379779
H	5.988706	8.911911	20.656710	H	3.379688	17.624806	24.503263
H	7.180740	9.762622	19.628976	H	3.306412	17.852786	26.266075
C	5.886137	5.004763	10.336246	H	4.805940	17.300442	25.496627
H	6.403437	4.067099	10.107510	C	10.229220	24.938669	18.402290
H	6.049816	5.227280	11.395439	H	9.580150	24.705217	17.551221
H	6.337686	5.802088	9.733285	H	11.080950	24.251991	18.376033
C	4.738490	10.575754	19.036194	H	10.616212	25.956224	18.270526
H	5.474877	11.396036	18.991908	C	17.256594	17.546196	17.508819
H	4.316843	10.544471	20.055101	H	17.604392	18.399522	16.901791
C	-0.367622	8.050685	12.169252	H	17.872300	16.667352	17.252358
H	-1.163802	8.030889	12.921587	C	10.844383	16.668453	19.264594
H	-0.683866	7.438803	11.318359	C	14.001187	19.905892	18.477942
H	-0.260623	9.083752	11.818335	H	14.991828	19.930321	18.031955
C	-1.496897	0.975627	18.071742	C	12.145448	12.826032	18.999383
H	-0.549400	1.212411	18.567192	H	12.832943	11.969518	18.890599
H	-2.114807	0.417953	18.783649	H	11.129079	12.503328	18.723009
H	-2.014624	1.915008	17.847545	C	14.118126	16.773860	15.744837
C	5.101184	10.436371	12.748950	H	13.492207	17.505915	16.283324
H	5.862742	11.234754	12.759707	H	13.842471	16.801184	14.676898
H	4.334940	10.687442	11.998270	C	13.468945	20.255062	22.750324
C	3.824839	11.497019	14.442454	H	14.490730	19.874414	22.628968
H	3.044157	11.765806	13.709860	H	13.213727	20.181031	23.813181
H	4.528589	12.344158	14.520561	H	13.466880	21.312422	22.466716
C	1.373108	8.448845	13.936504	C	15.574847	17.139230	15.878400
H	1.525439	9.476195	13.580445	H	16.203344	16.355075	15.424723
H	2.311366	8.104812	14.378989	H	15.761124	18.088720	15.348957
H	0.599685	8.458779	14.712275	C	8.497344	15.119999	25.241972
C	-0.579093	-1.180648	17.228815	H	9.264691	14.785424	25.949616
H	-0.444291	-1.848649	16.372461	H	7.954159	14.234154	24.895081
H	-1.172974	-1.708327	17.984961	H	7.795394	15.760004	25.786912
H	0.410216	-0.969688	17.648606	C	3.114652	20.296960	25.137597
C	2.338087	4.690910	18.400112	H	3.356794	21.362956	25.065435
H	1.686639	5.103276	19.180844	H	2.521664	20.152879	26.047143
H	3.330915	4.509874	18.827449	H	2.487523	20.023474	24.282149
H	1.935283	3.724138	18.084414	C	12.124596	13.313580	20.424956
C	8.945369	6.861996	16.226447	H	11.464282	14.192419	20.502800
H	9.698392	7.654063	16.382218	H	11.728576	12.519357	21.078999
H	9.456830	5.939302	15.914442	C	10.126713	14.976098	23.320797
C	3.621977	10.829656	18.056671	H	10.472475	15.464670	22.403832
H	2.923402	9.976610	18.053566	H	9.678552	14.016184	23.039374

H	3.068042	11.729703	18.373551	H	10.994266	14.782732	23.965164
C	6.976938	7.660269	19.231032	C	13.522326	14.144179	22.146391
H	7.701230	7.341996	20.000130	H	13.123567	13.397569	22.853972
H	6.181528	6.898421	19.174943	H	12.921192	15.061612	22.250038
C	8.203215	6.587036	17.507619	C	14.960822	14.424024	22.494995
H	7.420465	5.831725	17.329350	H	15.030053	14.677156	23.565772
H	8.908420	6.194257	18.258042	H	15.576996	13.529808	22.304341

**4: -3922.687438121882 E_h**			
In	6.483577	12.681516	6.127059
Fe	8.487142	13.945666	6.963889
O	4.162628	12.841888	5.209244
O	6.880518	16.199545	5.987056
O	10.744638	15.699682	7.509997
N	4.999116	11.925804	7.556815
O	8.344497	13.118845	9.771921
O	10.193366	12.112153	5.435334
N	6.427476	12.280871	3.957579
C	3.203378	12.238501	6.012979
C	1.912248	12.089440	5.554302
C	5.171578	11.849056	1.871541
H	6.092134	11.630561	1.339441
C	1.377142	11.011772	7.666411
C	4.054295	12.497247	3.866981
C	0.988639	11.460812	6.400367
H	-0.028420	11.322041	6.059700
C	5.271141	12.210899	3.228118
C	2.703197	11.174046	8.078717
H	3.025310	10.817561	9.052529
C	2.759788	11.994564	1.933920
H	1.796842	11.888901	1.453403
C	2.812888	12.369419	3.283901
C	3.669447	11.774183	7.251629
C	0.394766	10.321348	8.621262
C	10.027020	11.549875	1.899299
H	10.964730	11.362138	1.382790
C	3.937417	11.749429	1.222181
C	1.599134	12.657197	4.168897
C	9.563265	12.848886	2.047888
H	10.145136	13.672056	1.642608
C	9.295752	10.491236	2.421411
H	9.668642	9.476625	2.307926
C	5.334006	11.833029	8.931444
C	6.270513	10.620342	10.799200
H	6.785772	9.747275	11.192813
C	7.486112	15.273284	6.361230
C	8.371779	13.119922	2.722390
C	5.264217	12.766734	11.159902
H	5.003030	13.578353	11.834925
C	7.638217	12.039305	3.255449
C	4.983226	12.892081	9.801764
C	9.845889	14.998320	7.297007

C	8.096092	10.715153	3.094355
C	0.313152	12.070738	3.586983
H	-0.539518	12.305297	4.231678
H	0.381944	10.983489	3.477426
H	0.103724	12.508883	2.606328
C	6.001017	10.698085	9.431864
C	9.460111	12.823897	5.989513
C	5.895950	11.637207	11.663428
H	6.115576	11.561229	12.725037
C	7.101802	14.952976	1.571266
H	7.762692	14.917943	0.695584
H	6.716933	15.975054	1.670089
H	6.256600	14.282138	1.389003
C	5.658784	8.271912	8.845489
H	5.868461	7.930365	9.866260
H	5.946138	7.469912	8.154770
H	4.578832	8.431883	8.755787
C	6.428063	9.561010	8.525463
H	6.168518	9.845337	7.499229
C	4.341840	14.170592	9.292284
H	4.259165	14.093081	8.203255
C	0.895968	8.898169	8.933493
H	0.995763	8.314788	8.011492
H	0.192099	8.381482	9.598268
H	1.873254	8.916016	9.425075
C	2.925375	14.357105	9.852821
H	2.290387	13.502613	9.599268
H	2.466700	15.262156	9.437173
H	2.944218	14.460540	10.944764
C	7.869492	14.545500	2.839946
H	7.162184	14.572017	3.674713
C	8.311172	13.403485	8.647355
C	5.220239	15.391997	9.597748
H	5.318998	15.553646	10.677481
H	4.777219	16.294224	9.159953
H	6.223231	15.265780	9.178513
C	-1.016514	10.208581	8.031866
H	-1.440519	11.194538	7.811886
H	-1.675851	9.713919	8.754213
H	-1.024040	9.615245	7.110786
C	8.983001	15.552082	3.143122
H	9.580864	15.228172	4.000734
H	8.543213	16.525197	3.386706
H	9.648473	15.695093	2.283334
C	1.432021	14.191263	4.295620
H	2.337756	14.651158	4.700653
H	0.596566	14.422904	4.966211
H	1.233721	14.629200	3.310640
C	7.943887	9.335247	8.595003
H	8.488351	10.251175	8.340631
H	8.248997	8.547607	7.896925

H	8.254079	9.031510	9.601212
C	7.320618	9.557207	3.691255
H	6.296253	9.902624	3.866771
C	3.926309	11.366467	-0.262958
C	0.300447	11.123941	9.932791
H	1.272454	11.199153	10.429597
H	-0.399350	10.641447	10.626830
H	-0.054478	12.141625	9.735386
C	4.622122	10.005607	-0.454065
H	5.668190	10.042628	-0.136636
H	4.599635	9.709851	-1.510559
H	4.119622	9.230611	0.135283
C	7.922805	9.172257	5.048843
H	7.323631	8.394608	5.537957
H	7.974892	10.037845	5.719528
H	8.948582	8.803315	4.929802
C	7.241953	8.340150	2.765025
H	8.221935	7.871818	2.618333
H	6.846813	8.619814	1.782671
H	6.578235	7.582275	3.196765
C	4.683115	12.440742	-1.067683
H	4.213984	13.421749	-0.933416
H	4.676716	12.194929	-2.137306
H	5.724949	12.523811	-0.743766
C	2.505790	11.258903	-0.830930
H	2.555442	10.985317	-1.891029
H	1.969957	12.211389	-0.755683
H	1.920967	10.490658	-0.312914

9. References for supporting information

s1 J. Hicks, P. Vasko, J. M. Goicoechea, S. Aldridge, *Nature* **2018**, *557*, 92-95.

s2 L. P. Griffin, M. A. Ellwanger, A. E. Crumpton, M. M. D. Roy, A. Heilmann, S. Aldridge, *Angewandte Chemie International Edition* **2024**, *63*, e202404527.

s3 J. Cosier, A. M. Glazer, *Journal of Applied Crystallography* **1986**, *19*, 105-107.

s4 A. Technologies, **2011**.

s5 G. Sheldrick, *Acta Crystallographica Section A* **2015**, *71*, 3-8.

s6 G. Sheldrick, *Acta Crystallographica Section C* **2015**, *71*, 3-8.

s7 O. V. Dolomanov, L. J. Bourhis, R. J. Gildea, J. A. K. Howard, H. Puschmann, *Journal of Applied Crystallography* **2009**, *42*, 339-341.

s8 F. Neese, *WIREs Computational Molecular Science* **2012**, *2*, 73-78.

s9 F. Neese, *WIREs Computational Molecular Science* **2022**, *12*, e1606.

s10 S. Grimme, A. Hansen, S. Ehlert, J.-M. Mewes, *The Journal of Chemical Physics* **2021**, *154*, 064103.

s11 J.-D. Chai, M. Head-Gordon, *Physical Chemistry Chemical Physics* **2008**, *10*, 6615-6620.

s12 E. Caldeweyher, C. Bannwarth, S. Grimme, *The Journal of Chemical Physics* **2017**, *147*, 034112.

s13 F. Weigend, R. Ahlrichs, *Physical Chemistry Chemical Physics* **2005**, *7*, 3297-3305.

s14 J. K. B. E. D. Glendening, A. E. Reed, J. E. Carpenter, J. A. Bohmann, C. M. Morales, P. Karafiloglou, C. R. Landis, F. Weinhold, **2018**.

s15 E. D. Glendening, C. R. Landis, F. Weinhold, *Journal of Computational Chemistry* **2019**, *40*, 2234-2241.

s16 T. Lu, F. Chen, *Journal of Computational Chemistry* **2012**, *33*, 580-592.

s17 R. Bianchi, G. Gervasio, D. Marabello, *Inorganic Chemistry* **2000**, *39*, 2360-2366.
